# Concentrated Solar Light Photoelectrochemical Water Splitting for Stable and High‐Yield Hydrogen Production

**DOI:** 10.1002/advs.202309548

**Published:** 2024-03-09

**Authors:** Wan Jae Dong, Zhengwei Ye, Songtao Tang, Ishtiaque Ahmed Navid, Yixin Xiao, Bingxing Zhang, Yuyang Pan, Zetian Mi

**Affiliations:** ^1^ Department of Electrical Engineering and Computer Science University of Michigan 1301 Beal Avenue Ann Arbor MI 48109 USA

**Keywords:** concentrated solar light, hydrogen evolution, photoelectrochemical water splitting, stability

## Abstract

Photoelectrochemical water splitting is a promising technique for converting solar energy into low‐cost and eco‐friendly H_2_ fuel. However, the production rate of H_2_ is limited by the insufficient number of photogenerated charge carriers in the conventional photoelectrodes under 1 sun (100 mW cm^−2^) light. Concentrated solar light irradiation can overcome the issue of low yield, but it leads to a new challenge of stability because the accelerated reaction alters the surface chemical composition of photoelectrodes. Here, it is demonstrated that loading Pt nanoparticles (NPs) on single crystalline GaN nanowires (NWs) grown on *n^+^‐p* Si photoelectrode operates efficiently and stably under concentrated solar light. Although a large number of Pt NPs detach during the initial reaction due to H_2_ gas bubbling, some Pt NPs which have an epitaxial relation with GaN NWs remain stably anchored. In addition, the stability of the photoelectrode further improves by redepositing Pt NPs on the reacted Pt/GaN surface, which results in maintaining onset potential >0.5 V versus reversible hydrogen electrode and photocurrent density >60 mA cm^−2^ for over 1500 h. The heterointerface between Pt cocatalysts and single crystalline GaN nanostructures shows great potential in designing an efficient and stable photoelectrode for high‐yield solar to H_2_ conversion.

## Graphical abstract

Photoelectrodes consisting of Pt nanoparticles (NPs) loaded on single crystal GaN nanowires grown on *n^+^‐p* Si wafers reveal high hydrogen evolution activity, productivity, and stability under concentrated solar light. Lattice alignment between Pt and GaN at the heterointerface stably anchors the cocatalysts during the harsh reaction conditions. Repeated redeposition of Pt NPs greatly improves the performance and stability for over 1500 h.

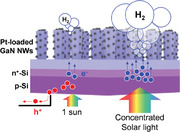

## 1 Introduction

Hydrogen (H_2_) stands as a clean energy source that can be produced through solar water splitting, offering a sustainable alternative to carbon‐emitting fossil fuels.^[^ ^1^ ^]^ Over time, numerous semiconductor photoelectrodes have harnessed solar energy for the production of green H_2_ through photoelectrochemical (PEC) water splitting.^[^ ^2^ ^]^ The photoelectrodes operating under solar light offer a voltage saving when compared to electrocatalysts operating in dark.^[^ ^3^ ^]^ However, unlike the electrochemical reactions,^[^ ^4^ ^]^ the maximum photocurrent density (J_ph_) is inherently constrained by the quantity of photogenerated charge carriers within the semiconductors, leading to limited H_2_ production yield. This issue can be addressed by illuminating concentrated solar light which can increase the number of photogenerated charge carriers, J_ph_, and H_2_ production rate. This has been demonstrated by integration of photovoltaic‐electrocatalyst (PV‐EC) devices,^[^ ^5^ ^]^ offering the added benefit of reducing costs associated with catalysts, semiconductor light absorbers, and electricity.^[^ ^6^ ^]^ Despite the advancements in PEC systems for H_2_ production, there has been no detailed study on the performance and long‐term stability of a monolithic photoelectrode capable of simultaneous light absorption, electron excitation, charge carrier separation, and catalyzing the hydrogen evolution reaction (HER) under concentrated solar light. Therefore, fundamental studies are required to better understand the processes involved in PEC HER and to develop more efficient and stable photoelectrodes that can unlock the full potential of concentrated solar energy.

Photoelectrodes have been typically fabricated by applying cocatalysts onto semiconductor materials.^[^ ^7^ ^]^ Up to date, high‐efficiency semiconductor materials (Si and III‐V semiconductors) for PEC water splitting in the aqueous solution showed poor stability due to photocorrosion.^[^ ^8^ ^]^ Therefore, passivation layers of amorphous oxides such as Al_2_O_3_ or TiO_2_ have been deposited on the semiconductors prior to applying the Pt nanoparticles (NPs) cocatalysts.^[^ ^9^ ^]^ While Pt cocatalysts show high activity for HER, their low adhesion has caused instability and limited the long‐term functionality of photoelectrodes.^[^ ^10^ ^]^ To mitigate this concern, researchers have explored solutions such as the use of reduced graphene oxide binder^[^ ^11^ ^]^ or metal oxide overlayer^[^ ^2^, ^12^ ^]^ to encapsulate Pt NPs, thereby preventing their detachment. However, this approach, while enhancing the stability of cocatalysts, tends to block active sites and hinder the mass transfer of reactants and products. In a recent study, a hydrogel protective layer was employed for Pt/TiO_2_/Sb_2_Se_3_ photocathode, resulting in an improvement in stability.^[^ ^13^ ^]^ The hydrogel protector prevented the agglomeration and detachment of Pt NPs and suppressed the photocorrosion of the TiO_2_ passivation layer. This finding highlighted the importance of both the mechanical and chemical considerations when designing a photoelectrode. Although protective schemes have resolved stability problems to some extent, there is still a great demand for innovative strategies to anchor Pt cocatalysts onto photoelectrodes to achieve efficient and stable PEC water splitting since this problem may become more significant when concentrated solar light is used to accelerate the H_2_ production rate.

In this study, we present a stable and efficient method for the production of high‐yield H_2_ using Pt nanoparticles (NPs)‐decorated GaN nanowires (NWs) grown on *n^+^‐p* Si photoelectrode. Under concentrated solar light at 6.4 sun (640 mW cm^−2^), the pristine Pt/GaN/Si exhibited high photocurrent density at 0 V versus reversible hydrogen electrode (V_RHE_) (J_0_) over 100 mA cm^−2^, but degraded within 0.5 h and stabilized thereafter. The rate of performance degradation was significantly accelerated under concentrated solar light compared to that observed under conventional 1 sun (100 mW cm^−2^) illumination. Surface chemical and microstructure analysis unveiled that the concentrated solar light induced rapid surface modifications and removed Pt NPs meanwhile some Pt cocatalysts having an epitaxial relation with GaN NWs were strongly anchored on the surface and remained even after vigorous H_2_ gas evolution. This finding guided us to redeposit Pt NPs on the reacted surface of photoelectrode, where more anchoring sites for Pt NPs were available, leading to enhanced HER activity and stability. This work identifies the stable bonding form of Pt NPs on single crystalline GaN NWs and elucidates strategies to obtain an efficient and durable photoelectrode working under concentrated solar light.

## 2 Results and Discussion

### 2.1 Concentrated Solar Light Photoelectrochemical Water Splitting

**Figure 1:**
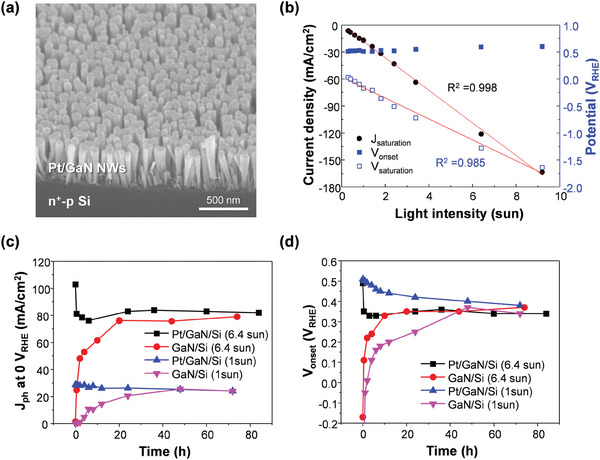
a) Tilt‐view SEM image of Pt‐loaded GaN nanowires / *n^+^‐p* Si photoelectrode. b) Plots of saturated current density (J_saturation_), onset potential (V_onset_), and saturation potential (V_saturation_) with light intensity. c) Photocurrent density at 0 V_RHE_ (J_0_) and d) V_onset_ for Pt/GaN/Si and GaN/Si measured as a function of time under 1 sun and 6.4 sun light.

Pt/GaN/Si photoelectrode was fabricated by vertical growth of *n‐*type GaN NWs on planar *n^+^‐p* Si wafer followed by photodeposition of Pt NPs as described in the supporting information experimental section. GaN NW arrays have a length of ≈400 nm, as observed in scanning electron microscopy (SEM) image (**Figure** **1**a). We demonstrated the concentrated solar light PEC water splitting using Pt/GaN/Si photoelectrode in an H‐type flow cell consisting of Pt wire counter electrode, and Ag/AgCl reference electrode with Nafion proton exchange membrane (Figure S1, Supporting Information). 0.5 m H_2_SO_4_ aqueous electrolyte was continuously circulated, and AM 1.5 G‐filtered solar light was irradiated on the backside of the photoelectrode during the reaction. When the light illuminates the *n^+^‐p* Si, the photoexcited electrons in the conduction band of Si drift toward *n*‐type GaN NWs due to the built‐in potential generated at the p‐n junction, whereas the photogenerated holes in the valance band of *p*‐Si move to the Cu back contact through GaIn eutectic alloy (Figure S2, Supporting Information).^[^ ^14^ ^]^ Since there is a negligible energy barrier between the conduction bands of *n*‐Si and *n*‐GaN, photogenerated electrons can efficiently migrate to the Pt/GaN surface and participate in the HER.

For the concentrated solar light irradiation, light intensity was controlled from 0.3 sun (30 mW cm^−2^) to 9.2 sun (920 mW cm^−2^) by varying the distance between the light source and the photoelectrode (Figure S3, Supporting Information). We first confirmed the need for a liquid flow system to provide fresh reactant and remove the gaseous H_2_ product during the vigorous reaction and found that a flow rate of 4 ml min^−1^ is required to each cathodic and anodic compartment to maintain high J_ph_ > 70 mA cm^−2^ (Figure S4, Supporting Information). Linear sweep voltammetry (LSV) curves of the Pt/GaN/Si photoelectrode exhibited a positive onset potential (V_onset_) > 0 V_RHE_, an increase in J_ph_ at the mid‐potential region, and a saturated photocurrent density (J_saturation_) at large biases (Figure S5, Supporting Information). J_saturation_ gradually increased from 6.5 to 163.6 mA cm^−2^ as solar power intensified from 0.3 to 9.2 sun. The linear correlation between J_sautration_ and light intensity indicates that the number of charge carriers generated in the photoelectrode, determined by the light intensity, is the main limiting factor for the J_ph_ (Figure 1b). The saturation potential (V_sautration_) at which the current density begins to saturate also linearly increased from 0.03 to −1.64 V_RHE_ with the light intensity. In contrast, the V_onset_ revealed a value of 0.51 – 0.60 V_RHE_ regardless of the light intensity.

With the light projection area of 0.13 cm^2^ (Figure S6, Supporting Information), LSV curves of Pt/GaN/Si were measured after each period of reaction time at 0 V_RHE_ under 1 sun light (Figure S7a, Supporting Information). As the reaction time increased from 0 to 72 h, V_onset_ gradually shifted to negative values and J_0_ showed a decreasing trend. This was because Pt NPs detached from the GaN surface. As the number of Pt NPs on the photoelectrode decreases, the charge carrier diffusion length that the photoelectrons should travel in the GaN NWs before reaching to the Pt NPs increases, resulting in recombination and decreased efficiency. In contrast, GaN/Si showed a gradual positive shift of V_onset_ and an increase in J_0_ with the progress of the reaction (Figure S7b, Supporting Information). This self‐improvement is known to be due to the partial substitution of N on the nonpolar crystal facets of GaN NWs with O during the reaction, leading to gallium oxynitrides formation and an enhancement of the catalytic properties for H_2_ evolution.^[^ ^15^ ^]^ It should be noted that the performance of Pt/GaN/Si rapidly degraded during the first 0.5 h reaction under 6.4 sun light and stabilized thereafter (Figure S7c, Supporting Information), whereas GaN/Si showed much faster self‐improvement during the initial 20 h and then stabilized (Figure S7d, Supporting Information). J_0_ (Figure 1c) and V_onset_ (Figure 1d) were plotted as a function of reaction time. Pt/GaN/Si showed an initial drop of J_0_ from 103 to 81 mA cm^−2^ and V_onset_ from 0.50 to 0.35 V_RHE_ in 0.5 h of concentrated solar light PEC HER whereas Pt/GaN/Si measured under 1 sun light revealed no degradation of J_0_ and much slower degradation speed of V_onset_. In the case of GaN/Si photoelectrode, the self‐improvement rate of J_0_ and V_onset_ is also faster under concentrated solar light compared to 1 sun light. The trend of applied bias photon‐to‐current efficiency (ABPE) changes over time was similar to the V_onset_ (Figure S8, Supporting Information). Overall, the in‐situ surface modifications such as Pt detachment and gallium oxynitride formation were accelerated under concentrated solar light, which largely deviate the performance and stability of photoelectrode from conventional measurement under 1 sun (100 mW cm^−2^).

### 2.2 Change in Surface Chemical Composition and Microstructure During the Reaction

**Figure 2:**
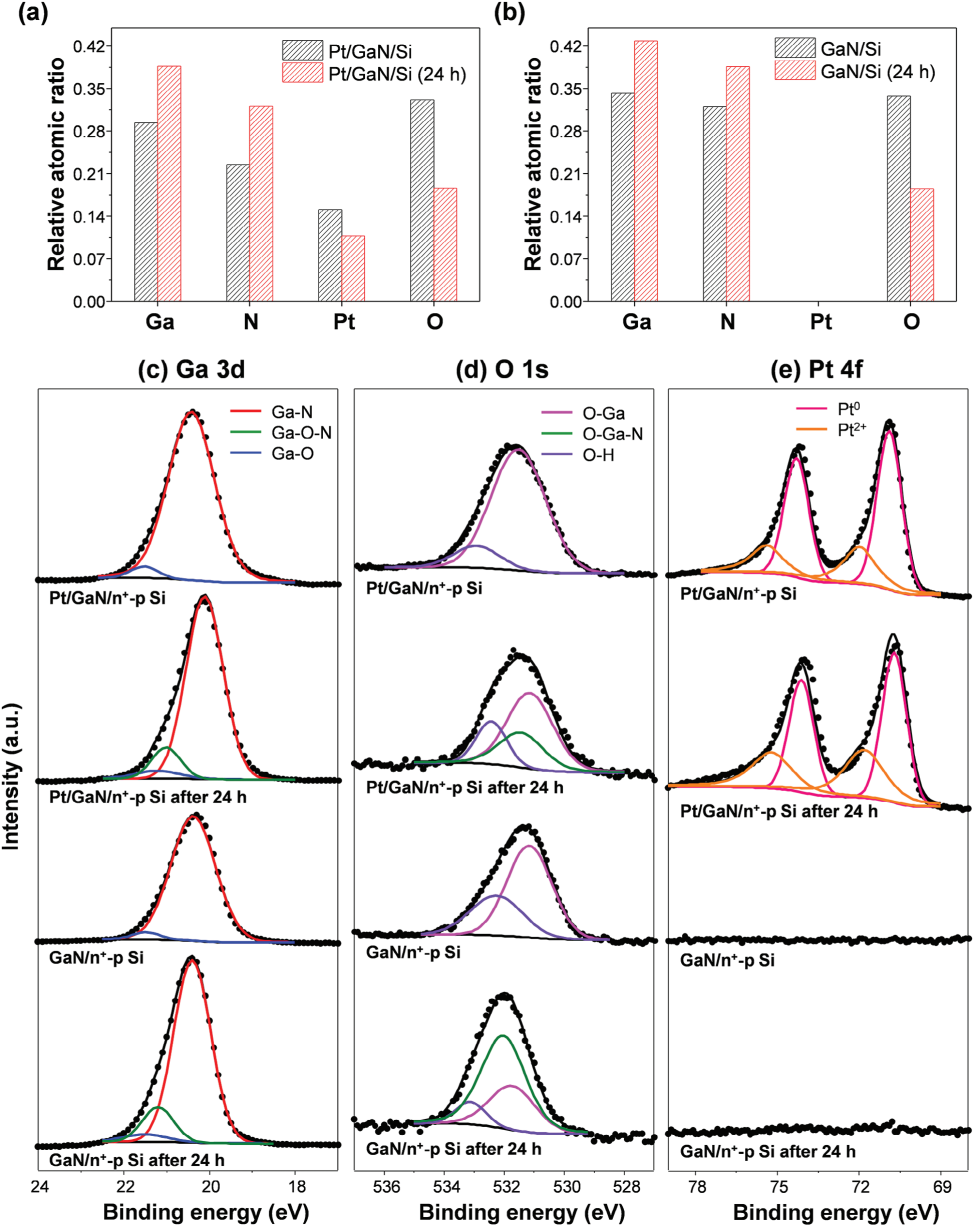
AR‐XPS analysis of Pt/GaN/Si and GaN/Si before and after reaction for 24 h under 6.4 sun light. Relative surface atomic ratio of a) Pt/GaN/Si and b) GaN/Si. XPS spectra of c) Ga 3d, d) O 1s, and e) Pt 4f.

To investigate the surface chemical composition of the photoelectrodes, angle‐resolved X‐ray photoelectron spectroscopy (AR‐XPS) was performed by varying the take‐off angle (TOA) of photo‐emitted electrons from the samples. The relative atomic ratio of pristine Pt/GaN/Si and the sample after the reaction for 24 h was measured (Figures S9a, and S9b, Supporting Information), and the values at TOA = 60° were compared (**Figure** **2**a). The surface atomic ratio of Ga and N increased after the reaction, while those of Pt and O reduced. This suggests that during the concentrated solar light PEC HER in the acidic electrolyte, some of the Pt NPs detached and gallium oxide (GaO_x_) species were removed from the surface. Consequently, more GaN was exposed to the outermost surface with a reduced number of Pt NPs. Similarly, GaN/Si also displayed an increased Ga and N content and reduced O ratio after the reaction under 6.4 sun light (Figure 2b; Figures S9c,d, Supporting Information). This provides further confirmation of the dissolution of the surface oxide layer. It is worth noting that no Pt 4f signals were detected on GaN/Si photoelectrodes, indicating that the self‐improvement of HER performance was not due to Pt contamination.

To gain further insight into the surface binding states, core‐level XPS spectra were analyzed for both pristine and reacted Pt/GaN/Si and GaN/Si photoelectrodes (Figures S10–S13, Supporting Information) and then Ga 3d, O 1s, and Pt 4f XPS spectra at TOA = 60° were plotted (Figure 2c–e). The Ga 3d XPS spectra of both pristine Pt/GaN/Si and GaN/Si showed a major peak of Ga‐N bond and a minor peak of Ga─O bond (Figure 2c). After the PEC HER reaction for 24 h, a new peak corresponding to Ga─O─N bond appeared, indicating the formation of gallium oxynitrides.^[^ ^15a,c^ ^]^ The O 1s spectra also showed emerging peaks of O─Ga─N in both Pt/GaN/Si and GaN/Si after the reaction, further confirming the formation of gallium oxynitride (Figure 2d). The Pt 4f spectra of pristine and reacted Pt/GaN/Si showed similar binding states of Pt^0^ and Pt^2+^ (Figure 2e).

**Figure 3:**
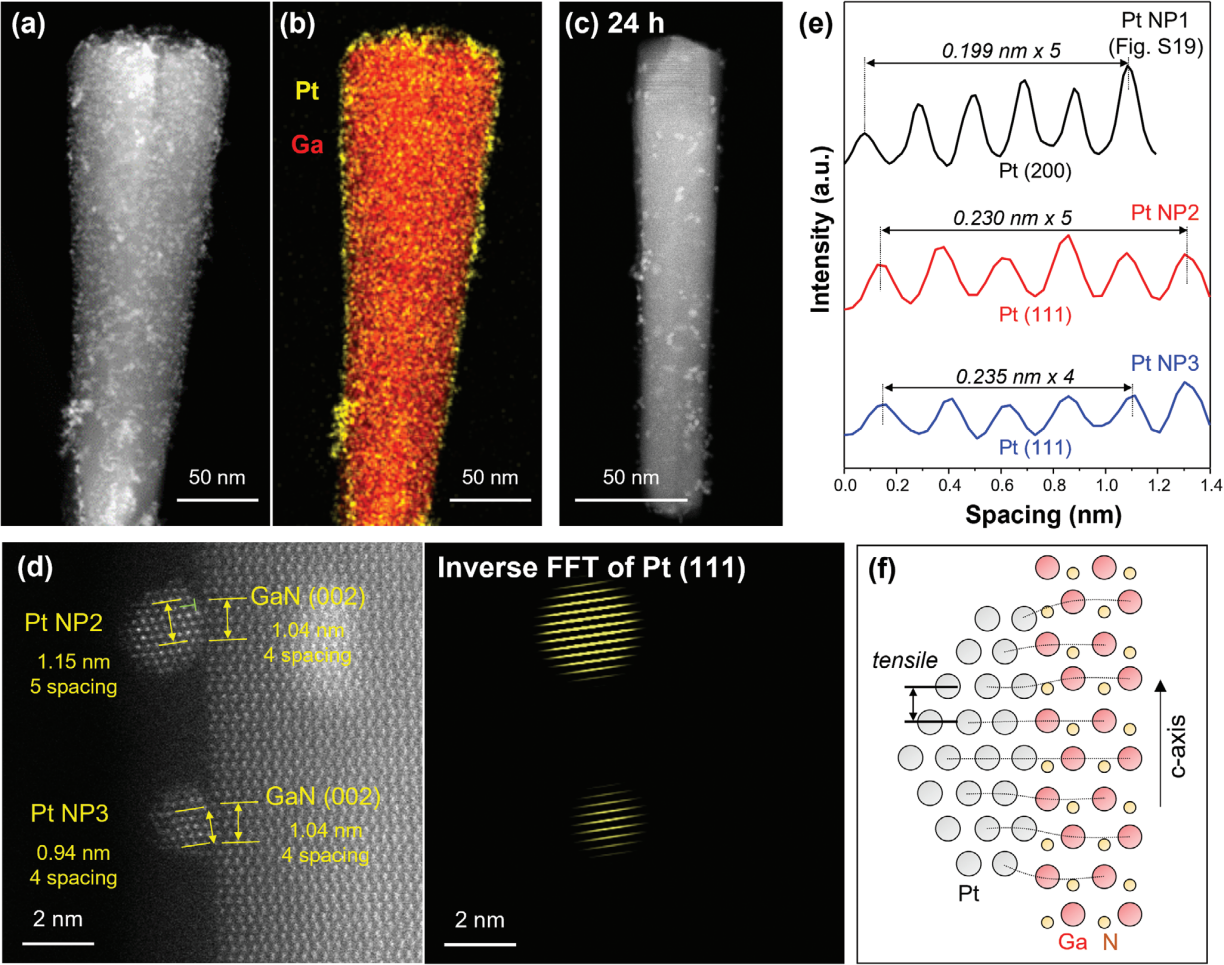
a) Low‐magnification HAADF‐STEM image and b) EDS elemental map of Pt/GaN/Si before the reaction. c) HAADF‐STEM image after 24 h reaction under 6.4 sun light. d) High‐resolution STEM image of Pt/GaN interface and inverse Fourier‐filtered image by masking Pt (111). Dislocations in Pt NPs are indicated in a green mark. e) Plots of d‐spacing of three Pt NPs in Figure 4d and Figure S19a (Supporting Information). f) Schematic lattice alignment at the Pt/GaN interface.

Microstructure of Pt/GaN/Si was examined using high‐angle annular dark‐field scanning transmission electron microscopy (HAADF‐STEM). The analysis revealed the presence of numerous Pt NPs with bright contrast on the top and sidewalls of GaN NW before the PEC HER (**Figure** **3**a; Figures S14, and S15, Supporting Information). Energy‐dispersive X‐ray spectroscopy elemental map showed dense and uniform distribution of Pt NPs on GaN NWs (Figure 3b). More interestingly, some Pt NPs with (111) and (200) orientations exhibited lattice alignment with GaN (002) planes (Figures S16 and S17, Supporting Information). After 24 h of reaction under concentrated solar light, a considerable number of Pt NPs were detached from the surface of GaN NWs, resulting in a significantly reduced and more sparse distribution of smaller sizes of Pt NPs on the GaN surface (Figure 3c; Figure S18, Supporting Information). In order to understand the mechanism behind the stable anchoring of Pt NPs to GaN NWs despite the vigorous H_2_ production, we conducted an analysis of the heterointerface between Pt NPs and GaN NWs at three different locations after the concentrated solar light PEC water splitting (Figure 3d; Figure S19, Supporting Information). Single crystal GaN NWs were grown along (002) orientation and Pt NPs with (200) and (111) crystal planes were found on the surface of GaN NWs. It is worth noting that the lattices of Pt NPs and GaN NWs were aligned with epitaxial relations. Specifically, the 5 lattice spacings of Pt (200) were aligned to the 4 lattice spacings of GaN (002), with an edge dislocation propagating in Pt NP1 (Figure S19a, Supporting Information). At another location, the lattices of Pt NP2 and Pt NP3 with (111) orientation exhibited an epitaxial relationship with GaN (002) (Figure 3d). Pt NP2 had a dislocation inside, while the adjacent Pt NP3 did not. In the case of a larger Pt NP4 (Figure S19b, Supporting Information), two dislocation lines were observed inside of the particle.

Upon comparing the lattice spacing of the three representative Pt NPs, Pt NP1 had a d‐spacing (0.199 nm) that was ≈ 1.4% elongated than the literature value of Pt (200), while Pt NP2 (0.230 nm) and Pt NP3 (0.235 nm) had d‐spacing extensions of ≈1.5% and 3.8%, respectively, compared to the literature value of Pt (111) (JCPDS 04–0802) (Figure 3e). Pt NPs with large sizes can form dislocations inside to release interfacial stress, while Pt NPs with small sizes show a preference for lattice expansion over dislocation formation (Figure 3f). This implies that the size of Pt NPs plays a crucial role in determining the strain relaxation behavior at the heterointerface. According to previous studies, it is known that tensile strain acting on Pt catalysts deteriorates their H_2_ evolution catalytic properties.^[^ ^16^ ^]^ This is likely one of the reasons why the Pt/GaN/Si photoelectrode degraded despite the presence of Pt NPs on the photoelectrode after the concentrated solar light experiment. Nevertheless, the lattice‐matched Pt NPs to GaN showed strong bonding strength, which allows them to maintain good stability even under harsh reaction conditions.

### 2.3 Improving the Stability of Pt NPs on GaN/Si Photoelectrode

**Figure 4:**
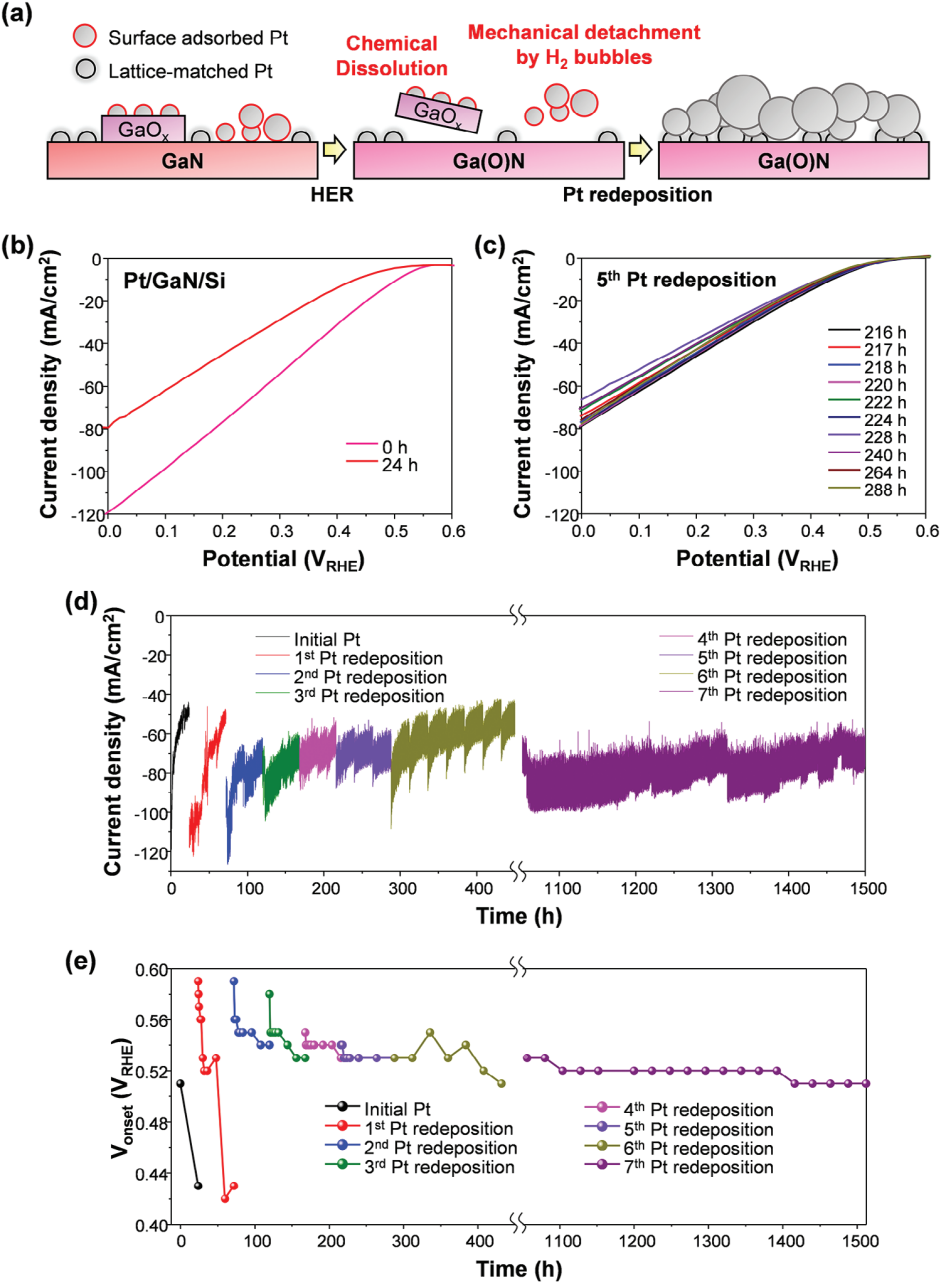
a) Schematic illustration of surface modification during the PEC HER. Surface adsorbed Pt NPs on GaN or GaO_x_ surface are removed while lattice‐matched Pt NPs on GaN are strongly anchored on the surface. Pt redeposition on the HER‐reacted Pt/GaN provides more stabilized Pt NPs. LSV curves of a) pristine Pt/GaN/Si (0 – 24 h) and b) after 5^th^ Pt redeposition (216 – 288 h). Plots of d) J_ph_ at 0 V_RHE_ and e) V_onset_ of Pt/GaN/Si over reaction time. The stability was tested over 1500 h.

We found that there were three different types of Pt NPs on the pristine GaN NWs: physically adsorbed Pt NPs, Pt NPs on surface oxides, and lattice‐matched Pt NPs with GaN NWs (**Figure** **4**a). However, during the concentrated solar light PEC HER, the acidic electrolyte dissolved the oxides with Pt NPs on it, and H_2_ bubbles caused mechanical detachment of the surface adsorbed Pt NPs, resulting in a small number of strongly anchored Pt NPs remaining on the surface and degrading the HER performance. To address this stability issue, we employed the redeposition of Pt NPs onto the reacted photoelectrodes, which has a higher possiblity of forming strong interactions between Pt NPs and the GaN lattices. Furthermore, regrowth of lattice‐matched Pt NPs can release the tensile strain, thus further improving both the HER activity and stability.

The comparison of LSV curves between pristine and 24 h‐reacted Pt/GaN/Si showed an evident degradation (Figure 4b). However, after the Pt redeposition, the regenerated Pt/GaN/Si recovered the high performance (Figure S20, Supporting Information). Interestingly, after the 5^th^ time of redeposition of Pt NPs, the stability dramatically improved (Figure 4c). Furthermore, there was no noticeable degradation of J_0_ after the 7^th^ Pt redeposition (Figure 4d). The degradation speed of V_onset_ (Figure 4e) and ABPE (Figure S21, Supporting Information) also slowed down after repeated Pt redeposition, indicating that the reacted surface provided more stable anchoring sites for Pt NPs. As a result, excellent performance and stability were achieved, with V_onset_ > 0.5 V_RHE_, J_ph_ > 60 mA cm^−2^, and high faradaic efficiency of H_2_ > 97% maintained over a period of 1500 h (Figure S22, Supporting Information and Movie S1).

After 5 times of Pt redeposition and 288 h long‐term stability test, there were a large number of Pt NPs remaining on the GaN surface (Figure S23, Supporting Information). TEM image revealed that Pt aggregates consist of agglomerated Pt NPs with void inside, indicating regrowth and an increase in the size of Pt NPs during the redeposition process. The lattice spacings of the Pt aggregates were found to be 0.227 nm, which is consistent with the literature value of Pt (111) (Figure S24, Supporting Information). These results confirm that the tensile strain in Pt NPs was released during the regrowth and explain why the V_onset_ stabilized at a value higher than 0.5 V_RHE_. The extent of tensile strain resulting from alignment at the heterointerface varies depending on the size of Pt NPs, with larger NPs leading to the release of tensile stress and an improvement in catalytic performance. This is a unique characteristic of the heterointerface between Pt NPs and GaN NWs because single‐crystal GaN has a well‐defined crystal structure that can potentially influence the behavior of Pt NPs. In contrast, previous studies have shown that Pt redeposition on an amorphous TiO_2_ passivation layer, which lacks a well‐defined crystal structure, resulted in a decrease in the J_ph_ even after the repeated Pt redeposition.^[^ ^17^ ^]^ Understanding how Pt cocatalysts adhere to the surface of conventional photoelectrodes has been challenging due to the limitations and complexities associated with the metal/amorphous oxide heterointerface. However, a clear understanding of the bonding mechanism between the cocatalyst and photoelectrode can be gained by using a single crystalline GaN NWs and forming Pt cocatalysts on them. In particular, the lattice alignment between Pt cocatalyst and GaN NWs can induce strong bonding strength, anchoring the Pt cocatalysts onto the photoelectrodes even under harsh concentrated solar light. Nevertheless, the utilization efficiency of Pt was still limited by the detachment of Pt cocatalysts from GaN NWs due to in‐situ surface modification during the initial reaction. To improve the utilization efficiency of Pt, it is essential to develop a new strategy, such as implementing surface treatment methods for GaN that closely mimic the post‐reaction surface. This will enable a strong binding at the interface between GaN and Pt, thereby minimizing the detachment of Pt from the surface.

In the case of irradiation with very strong solar light (40 sun), J_0_ could be increased to over 240 mA cm^−2^ (Figure S25, Supporting Information). However, the rubber O‐rings used in the flow cell were damaged by ultraviolet light and photothermic heat after ≈52 h. Therefore, the reactor will further be carefully designed to maintain a stable operation of concentrated solar light water splitting. Despite the stability issue caused by photo‐induced heat, it is noteworthy that the photothermal effect, where the absorption of light leads to localized heating, has the potential to further enhance the efficiency of reaction kinetics at locally elevated temperatures on the surface of photoelectrodes under concentrated solar light. While studies have explored gas‐phase photothermal catalytic reactions,^[^ ^18^ ^]^ the impact of photothermal effects on water‐splitting reactions in aqueous medium has been rarely investigated. Therefore, understanding the distribution of local high temperatures on the photoelectrode surface within the aqueous solution holds significant importance. The development of design strategies that harness synergistic photo and thermal energies can revolutionize the efficiency of hydrogen production compared to conventional PEC reactions.

In comparison to the Si‐based photoelectrodes under 1 sun light illumination, Pt/GaN/Si photoelectrodes under concentrated solar light (6.4 sun) exhibited ≈4‐fold higher H_2_ production rate and one order of magnitude higher J_0_ than the photoelectrodes of III‐V, oxides, or chalcogenides semiconductors (Table S1, Supporting Information). Up to date, various attempts have been made to use III‐nitride semiconductors for the application of PEC water splitting. While the previous works improved the efficiency and stability to some extent through engineering of III‐nitirde photoelectrodes,^[^ ^19^ ^]^ research at the interface between III‐nitride and its impact on stability is still unexplored, especially under concentrated solar light. Therefore, based on the performance variation of Pt/GaN/Si, the following protocol is proposed for evaluating the stability of PEC water splitting using concentrated solar light. The stability showed two distinct phases, influenced by accelerated surface reactions under concentrated solar light. Initially, there was a rapid drop in J_0_ during the early stage of the reaction (within the first 100 h) due to Pt detachment. Subsequently, the performance stabilized with the J_0_ dropping to ≈95% between 100 – 1500 h of operation. When photoelectrodes are exposed to concentrated solar light, the photocurrent density exhibits a linear increase, while other surface chemical and mechanical reactions that affect catalytic stability likely follow an exponential function. Therefore, in an accelerated reaction environment, a new approach for defining stability is necessary. By extrapolating the lifetime based on a period of stabilization (>100 h) and 5 h per day operation from the practical PEC reaction under daylight, we expect the photoelectrode to operate for more than 5 years while maintaining ≈80% of its initial J_0_ value. This suggests promising robustness for practical applications of the photoelectrode in concentrated solar light PEC systems.

Demonstration of stable photoelectrodes working under accelerated reaction conditions will not only reduce the material cost (light absorbers and cocatalysts) but also increase the hydrogen production rate per unit photoelectrode area, thus offering economic benefit as it has been studied through the PV‐EC systems. However, in contrast to PV‐EC systems, PEC reactions occur with semiconductor materials immersed in the aqueous electrolyte. Hence, the stability of the semiconductor materials becomes very significant. The stability advancements in this study will promote the developing wireless solar water‐splitting devices or artificial photosynthetic materials that operate under concentrated solar light in the aqueous solution. Furthermore, there is an opportunity to harness the photothermal effect on the surface of photoelectrodes. The localized heating induced by concentrated solar light can enhance reaction kinetics. This has the potential to bring significant improvements of efficiency and stability and realize low‐cost solar fuel production.

## 3 Conclusion

In summary, we demonstrated that heterointerface between Pt cocatalysts and single crystal GaN nanostructures holds great potential for developing an efficient, stable, and low‐cost photoelectrode for high‐yield solar to H_2_ conversion under concentrated solar light. Although there was rapid decay in the initial 0.5 h of reaction due to accelerated surface modifications, Pt/GaN/Si maintained high performance thereafter owing to the stably anchored lattice‐matched Pt NPs on GaN NWs. Furthermore, the redeposition of Pt NPs on the reacted surface significantly improved the stability of the photoelectrode, outperforming conventional photoelectrodes. The insights gained into the stable bonding form of Pt NPs on single crystalline GaN NWs and strategies for obtaining an efficient and durable photoelectrode have important implications for the development of sustainable energy technologies toward a greener future.

## Conflict of Interest

Some IP related to this work was licensed to NS Nanotech, Inc. and NX Fuels, Inc., which were co‐founded by Z. Mi. The University of Michigan and Mi have a financial interest in the companies.

## Supporting information

Supporting Information

Supporting Information

## Data Availability

The data that support the findings of this study are available from the corresponding author upon reasonable request.
