# Supplementary material for: Concentrated Solar Light Photoelectrochemical Water Splitting for Stable and High‐Yield Hydrogen Production
Source: Adv Sci (Weinh). 2024 Mar 9;11(26):2309548. doi: 10.1002/advs.202309548 (PMC11234434; doi:10.1002/advs.202309548)
Supplement: Supplementary file 1 — Supporting Information [file ADVS-11-2309548-s001.pdf]

ADVANCED SCIENCE
Open Access

Supporting Information

for *Adv. Sci.*, DOI 10.1002/advs.202309548

Concentrated Solar Light Photoelectrochemical Water Splitting for Stable and High-Yield Hydrogen Production

*Wan Jae Dong, Zhengwei Ye, Songtao Tang, Ishtiaque Ahmed Navid, Yixin Xiao, Bingxing Zhang, Yuyang Pan and Zetian Mi**

Supporting Information

Concentrated Solar Light Photoelectrochemical Water Splitting for Stable and High-Yield Hydrogen Production

Wan Jae Dong, Zhengwei Ye, Songtao Tang, Ishtiaque Ahmed Navid, Yixin Xiao, Bingxing Zhang, Yuyang Pan, Zetian Mi*

Department of Electrical Engineering and Computer Science, University of Michigan, 1301 Beal Avenue, Ann Arbor, MI 48109, USA

*E-mail address: ztmi@umich.edu

Experimental section

Growth of GaN nanowires on n^+-p Si wafer

n^+-p Si wafer was prepared by a standard thermal diffusion process using a 2-inch (100) Si wafer. Phosphorus, an n-type dopant, was spin-coated on the front side of the polished p-type Si (100) wafer. Boron, a p-type dopant, was spin-coated on the backside of the wafer. Then, the wafer was thermally annealed at 950 ºC under a nitrogen atmosphere for 4 h. Plasma-assisted molecular beam epitaxy was employed for the growth of GaN nanowires on the front side of the n^+-p Si wafer under nitrogen rich-condition with an N_2 flow rate of 1.0 standard cubic centimeter per minute. The substrate temperature was held at 790 ºC and the growth duration was ~2 h. The forward plasma power was 350 W with Ga flux beam equivalent pressure of 5×10^{-8} Torr.

Photodeposition of Pt cocatalyst

A quarter wafer of GaN/Si was placed on a Teflon holder and placed in the glass reactor with a quartz lid. 20 μl of 0.2 M chloroplatinic acid hydrate (99.9%, Sigma Aldrich), 55 ml of deionized water, and 11 ml methanol were filled into the glass reactor. The reactor was evacuated using a rotary vacuum pump for 5 min. Then, the light was irradiation on the sample through quartz lid using a 300 W xenon lamp for 30 min. The light absorbed by GaN nanowires generate electrons and holes in conduction and valance bands, respectively. The photogenerated electrons migrate to the GaN surface and reduce platinum ions derived from chloroplatinic acid hydrate in the aqueous solution, leading to the growth of Pt nanoparticles on GaN nanowires. To maintain charge balance, the photogenerated holes participate in the oxidation reaction of sacrificial methanol, which is introduced into the solution. Finally, after 30 minutes, Pt-coated GaN NWs were obtained, rinsed with DI water, and dried by blowing

air.

Preparation of back contact

A liquid GaIn eutectic alloy was applied to the Cu back contact with a circular hole in the center for the incident light to pass through. Then, the GaIn eutectic alloy was sandwiched between the Cu back contact and the backside of n^+-p Si wafer for ohmic contact.

Concentrated solar light photoelectrochemical measurements

The intense simulated solar light (0.3-9.2 sun) was illuminated on the backside of the photoelectrode through a hole in the center of Cu back contact and H_2 evolution reaction was conducted on the front side (Pt/GaN nanowires) in an H-type flow cell with Pt wire counter electrode and Ag/AgCl filled with 3 M KCl reference electrode. A Nafion proton exchange membrane was placed in the middle of the cathodic and anodic compartments to separate them. The Nafion membrane was pretreated by heating it in H_2O_2 (5%) aqueous solution at 80 °C for 1 h and deionized water at 80 °C for 1 h, respectively, followed by treatment in 0.05 M H_2SO_4 for 1 h and stored in deionized water. The electrolyte was an aqueous solution of 0.5 M H_2SO_4 prepared by diluting the concentrated sulfuric acid (Sigma-Aldrich) in deionized water. pH value (= 0.25) of the electrolyte was measured using a pH meter (Mettler Toledo). A potentiostat (Biologic SP-200) was used for the photoelectrochemical measurements. The measured potentials ($V_{Ag/AgCl}$) (V) were converted to the reversible hydrogen electrode (V_{RHE}) (V) by using the Nernst function: $V_{RHE} = V_{Ag/AgCl} + 0.197 + 0.0591 \times pH$. The light source used for the illumination was LCS-100 (ORIEL) and the light intensity with an air mass 1.5 global (AM 1.5G) filter was calibrated by adjusting the distance from the sample to the light source. In the case of highly concentrated solar light (40 sun), a 300 W xenon lamp with AM 1.5G

filter was used. The electrolyte was circulated by a Masterflex Ismatec microflow pump (Cole-Parmer) at a rate of 10 ml/min during the reaction. To quantify the H_2 product, the liquid in the cathodic reactor continuously circulated in the closed system connected to a sealed chamber and then the collected H_2 product in the sealed chamber was measured by a gas chromatograph (Shimadzu GC-8A). All measurements were conducted under ambient pressure at room temperature.

Redeposition of Pt cocatalyst

After completing each step of the photoelectrochemical hydrogen evolution reaction, the Pt/GaN/Si photoelectrode was removed from the reactor chamber for redeposition. The identical Pt photodeposition process described above was then carried out for a duration of 30 minutes in an aqueous solution composed of 20 μl of 0.2 M chloroplatinic acid hydrate, 55 ml of deionized water, and 11 ml of methanol under a 300 W Xe lamp. Subsequent to the photodeposition, the dried samples were reintroduced into the reactor for the evaluation of stability and performance.

Characterization

SEM analysis was conducted using MIRA3 TESCAN with an accelerating voltage of 10 kV. XPS was measured using a Kratos Axis Ultra XPS with a monochromatic Al Kα source. STEM and STEM-EDS images were collected at 200 kV using JEOL 2100F microscope with Cs-corrector.

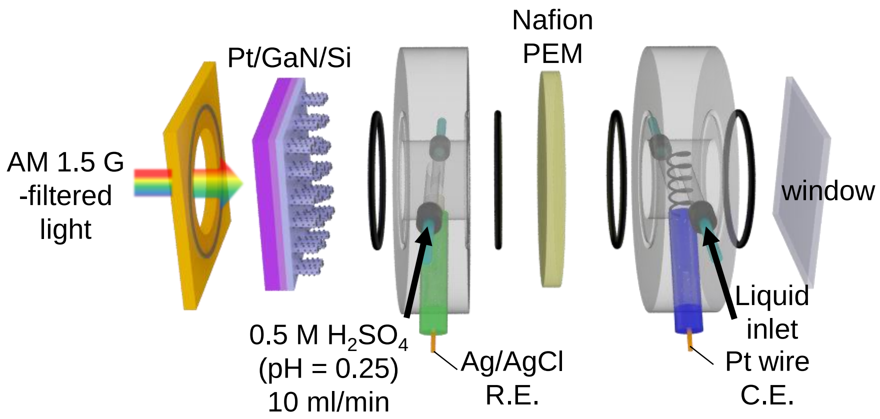

Figure S1. Schematic of H-type flow cell for concentrated solar light photoelectrochemical water splitting. Light illuminated from the backside of Si wafer. Pt wire counter electrode, Ag/AgCl reference electrode, and Nafion membrane were used with 0.5 M H_2SO_4 aqueous electrolyte.

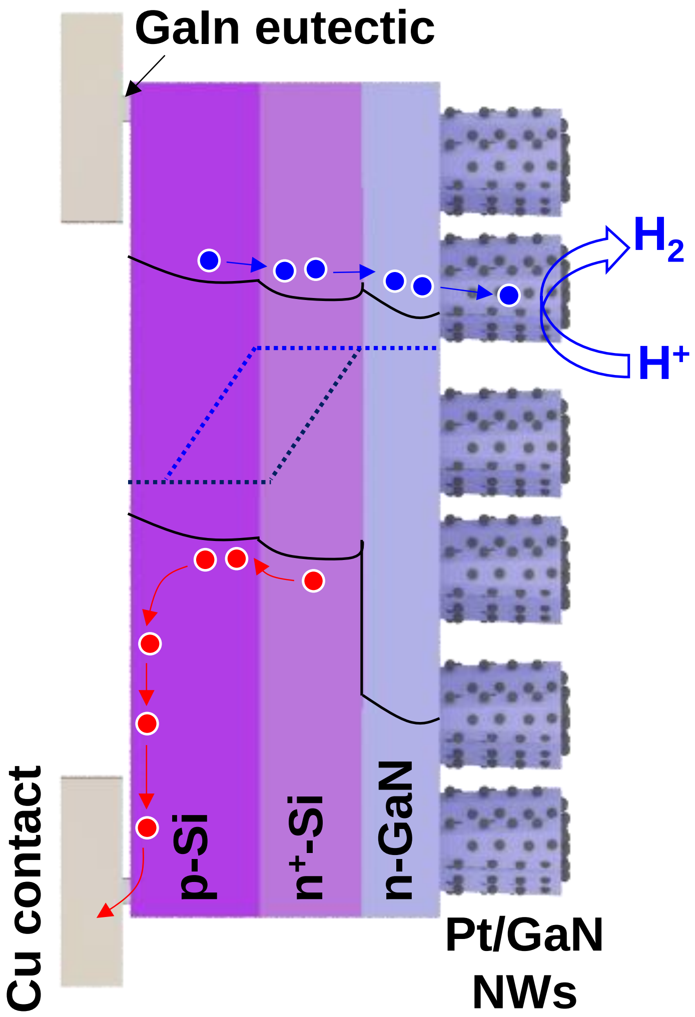

Figure S2. Schematic illustration of photoelectrode with conduction and valance band edge positions of each material. Photogenerated electrons in the conduction band of n^+-p Si can migrate to n-GaN without significant energy barrier and participate in hydrogen evolution reaction at the Pt/GaN surface. Photogenerated holes in the valance band of n^+-p Si laterally move toward the Cu back contact through GaIn eutectic alloy.

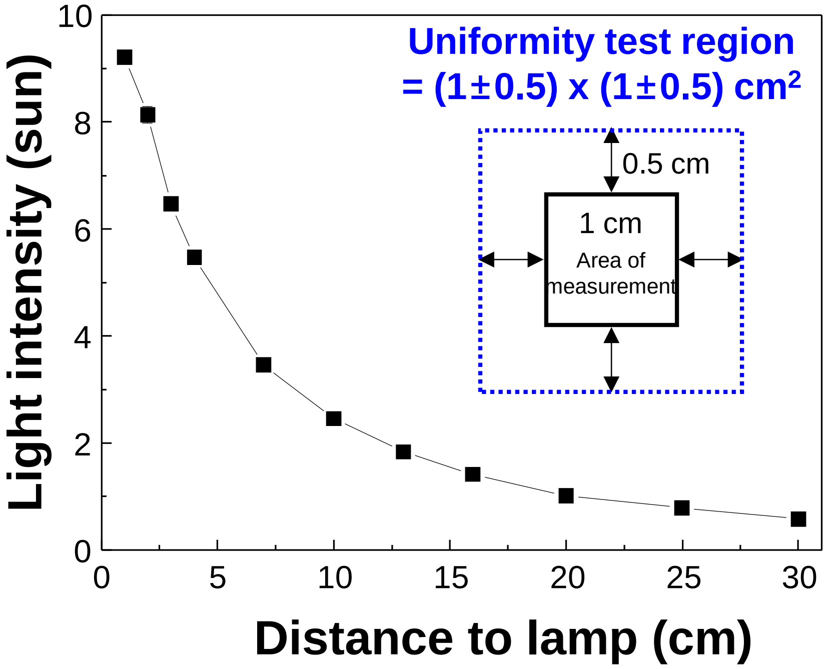

Figure S3. Calibration curve of light intensity with the distance between the photoelectrode and light source. The tested area was (1±0.5) × (1±0.5) cm^2.

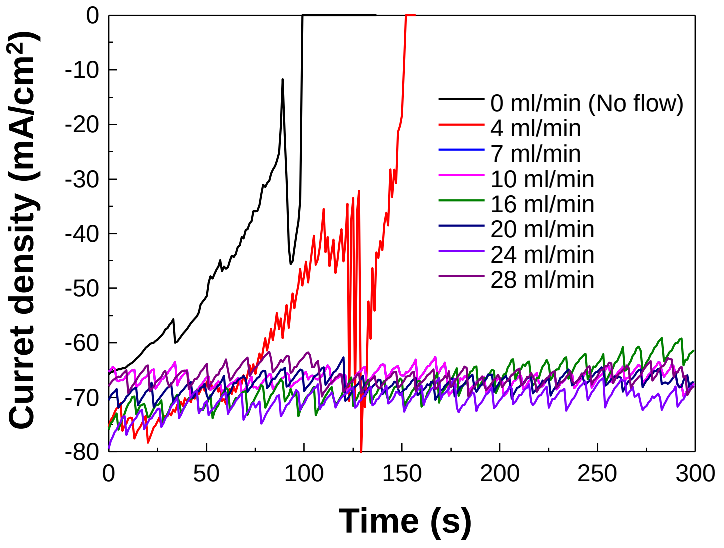

Figure S4. Chronoamperometric curves measured at -0.6 V_{RHE} under 6.4 sun light illumination with different liquid flow rates. Liquid flow rate was changed from 0 to 28 ml/min. Slow liquid circulation (0 and 4 ml/min) cannot remove H_2 gas bubbles formed on the photoelectrode surface, resulting in a rapid drop in photocurrent density. A flow rate of > 4 ml/min was required to retain the hydrogen evolution reaction in this condition.

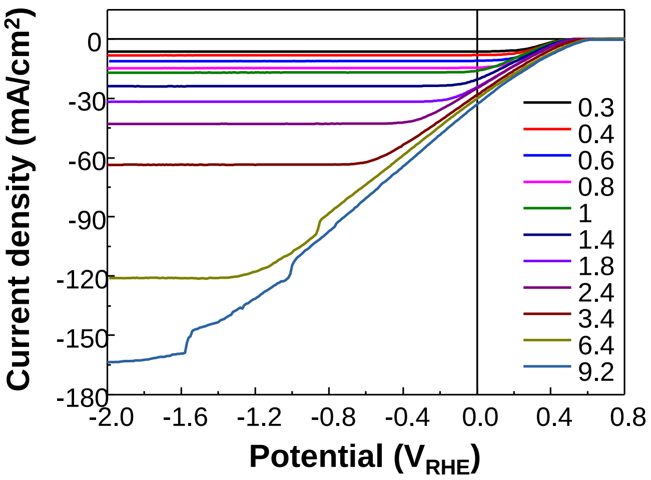

Figure S5. LSV curves of the photoelectrode under different light intensities (0.3 – 9.2 sun)

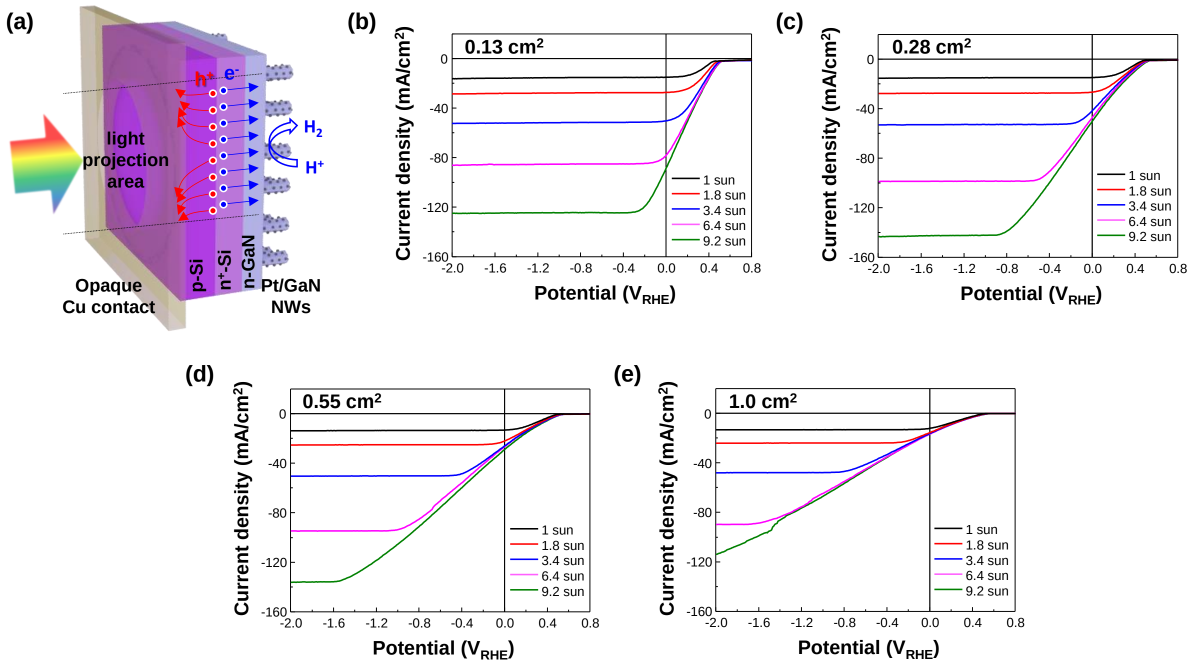

Figure S6. (a) Schematic illustration of charge carrier separation and H_2 evolution on Pt-loaded GaN NWs / n^{+}-p Si photoelectrode under light illumination. (b-e) Linear sweep voltammetry curves of photoelectrode with different light intensities. The light projection area varied from 0.13 to 1.0 cm^2. A smaller projection area resulted in a smaller $V_{saturation}$ and higher J_0.

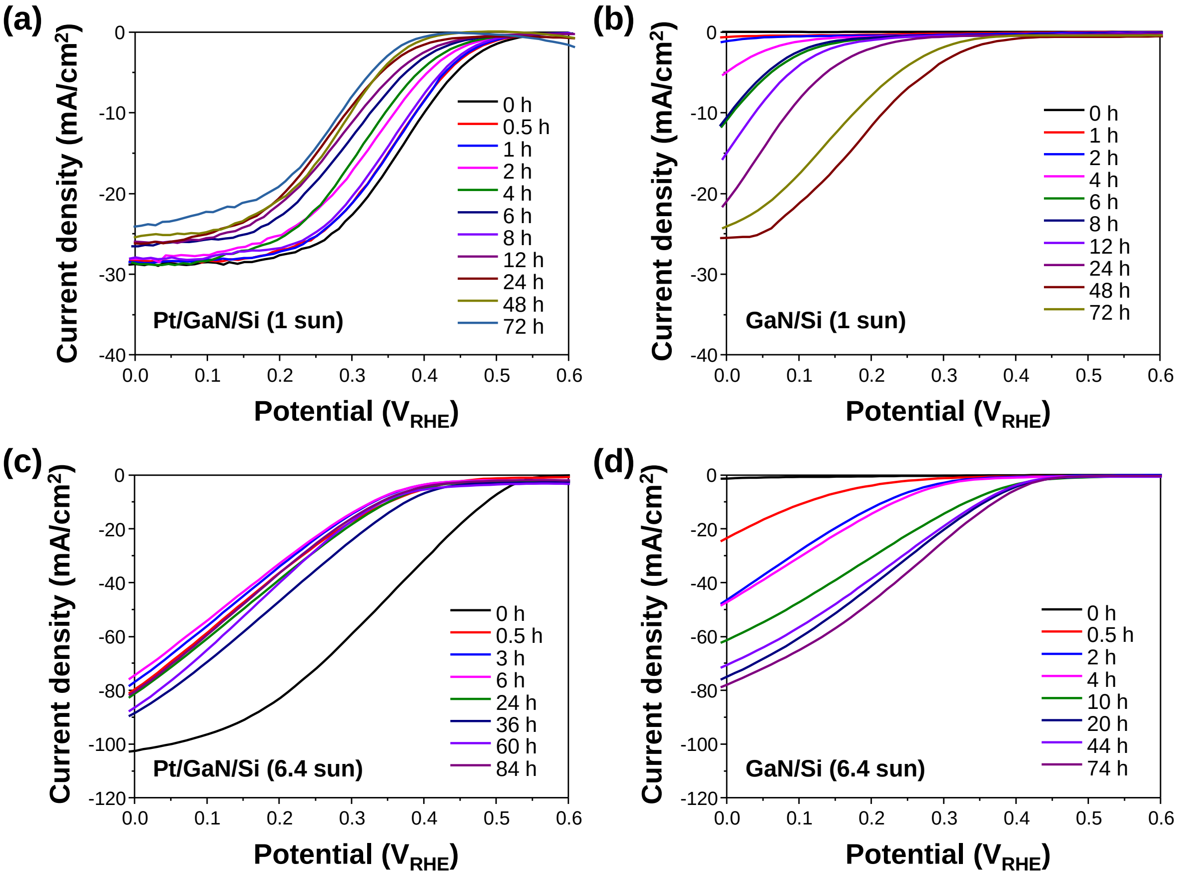

Figure S7. LSV curves of (a,c) Pt/GaN/Si and (b,d) GaN/Si photoelectrodes after each period of reaction. Light intensity used for measurement was 1 sun for (a,b) and 6.4 sun for (c,d).

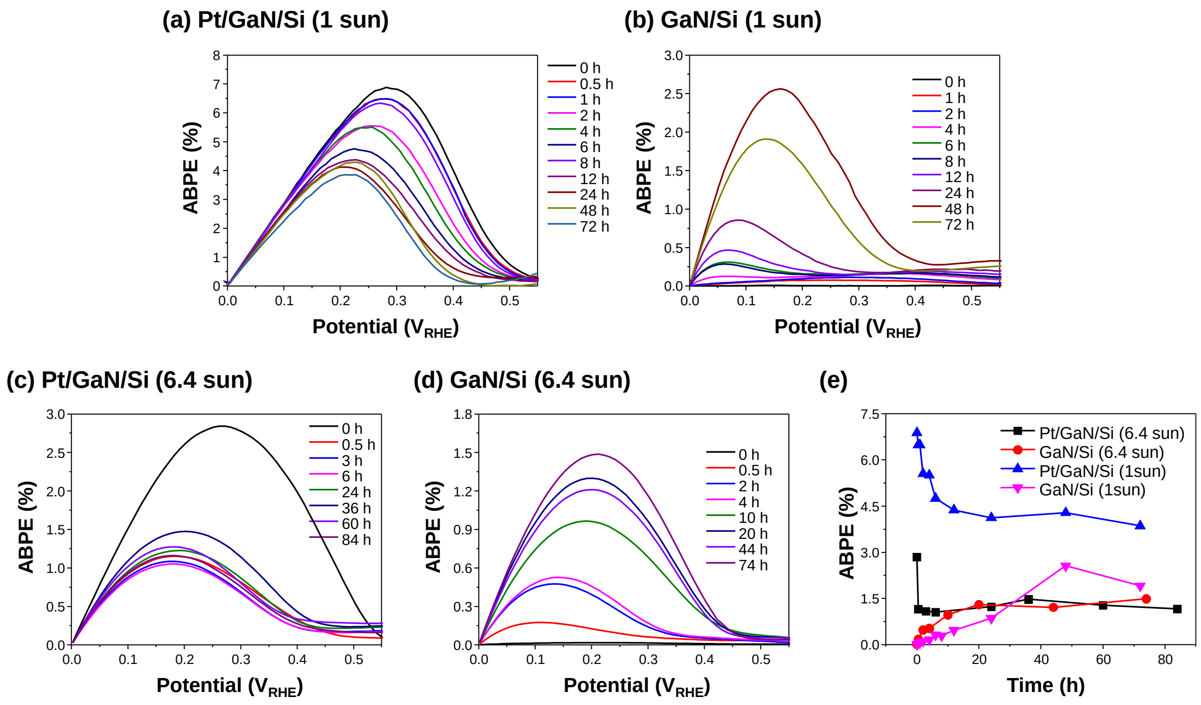

Figure S8. Applied bias photon-to-current efficiency (ABPE) of (a,c) Pt/GaN/Si and (b,d) GaN/Si photoelectrodes after each period of reaction. Light intensity used for measurement was 1 sun for (a,b) and 6.4 sun for (c,d). (e) Plots of maximum ABPE with reaction time for Pt/GaN/Si and GaN/Si measured under 1 sun and 6.4 sun light.

Compared to 1 sun illumination, the saturation photocurrent density under concentrated solar light linearly increased (Figure 1b). However, in the potential range above 0 V_{RHE}, where the photoelectrode exhibits its advantages over electrocatalytic hydrogen evolution, the increase in photocurrent density does not proportionally correlate with light intensity (Figure S7). Therefore, when the light intensity was increased from 1 to 6.4 suns, the ABPE of Pt/GaN/Si decreased from 6.9% (Figure S8a) to 2.8% (Figure S8c), respectively, despite the increase in photocurrent density and production yield of H_2. During the reaction, the ABPE of Pt/GaN/Si gradually decreased under both 1 and 6.4 sun light conditions. Meanwhile, the ABPE of GaN/Si under 1 and 6.4 suns was ~0%, but gradually increased to 1.9% (Figure S8b) and 1.5% (Figure S8d), respectively.

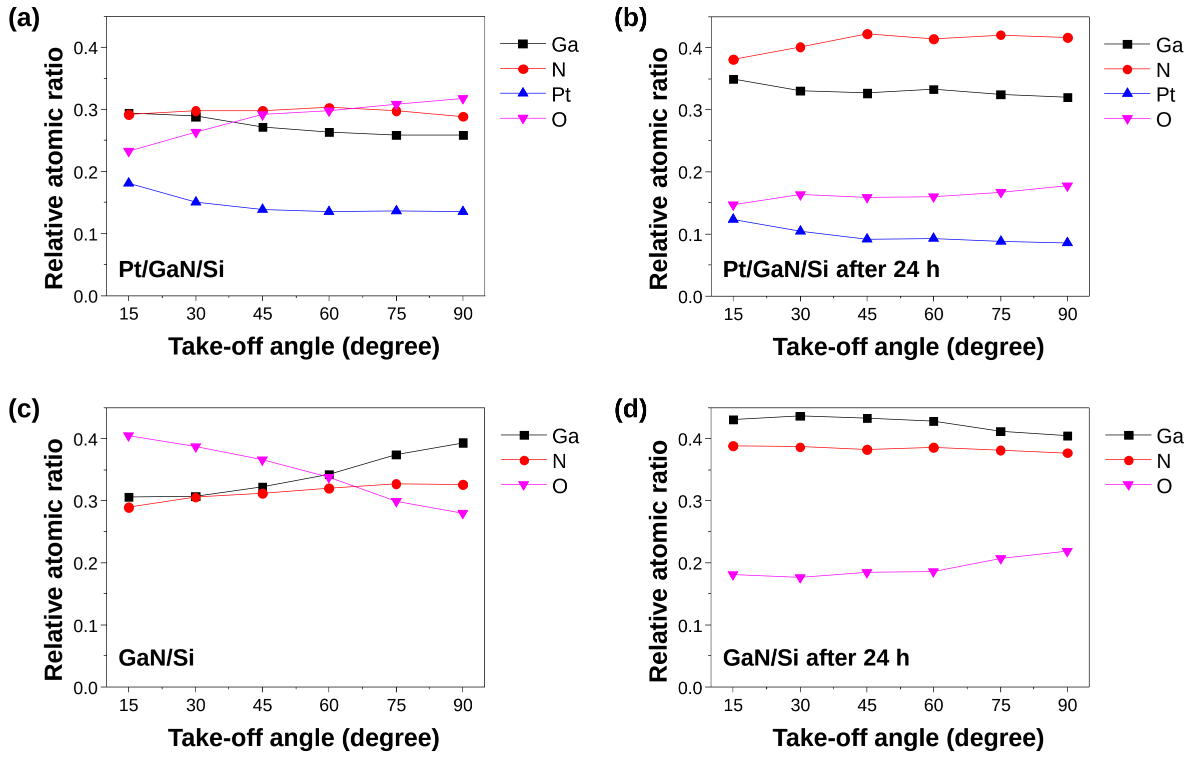

Figure S9. Relative atomic ratio analysed by angle-resolved X-ray photoelectron spectroscopy (AR-XPS) for (a) pristine Pt/GaN/Si, (b) Pt/GaN/Si after 24 h reaction, (c) pristine GaN/Si, and (d) GaN/Si after 24 h reaction. Reactions were performed at 0 V_{RHE} under 6.4 sun light.

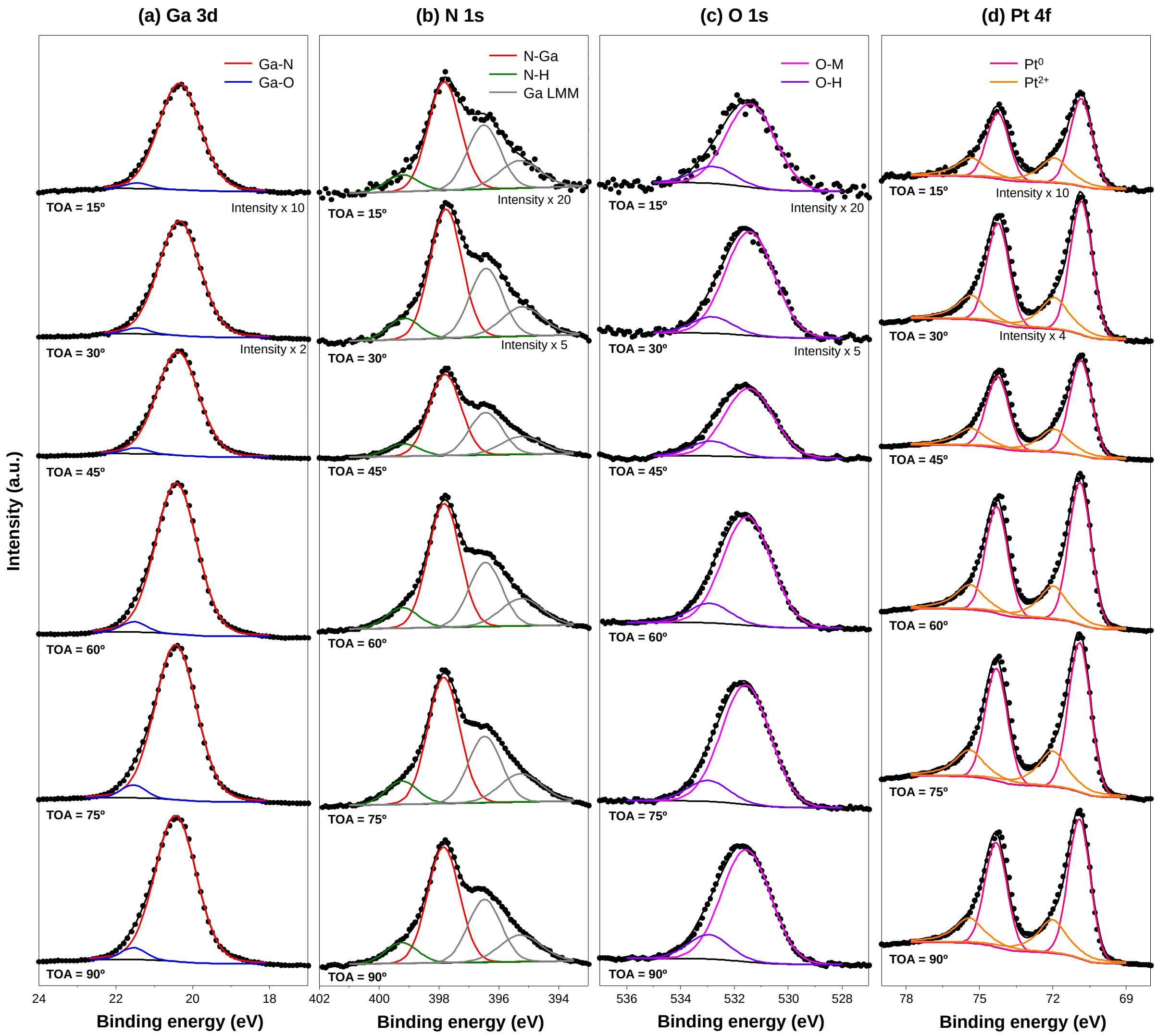

Figure S10. (a) Ga 3d, (b) N 1s, (c) O 1s, and (d) Pt 4f AR-XPS spectra of pristine Pt/GaN/Si with different take-off angles.

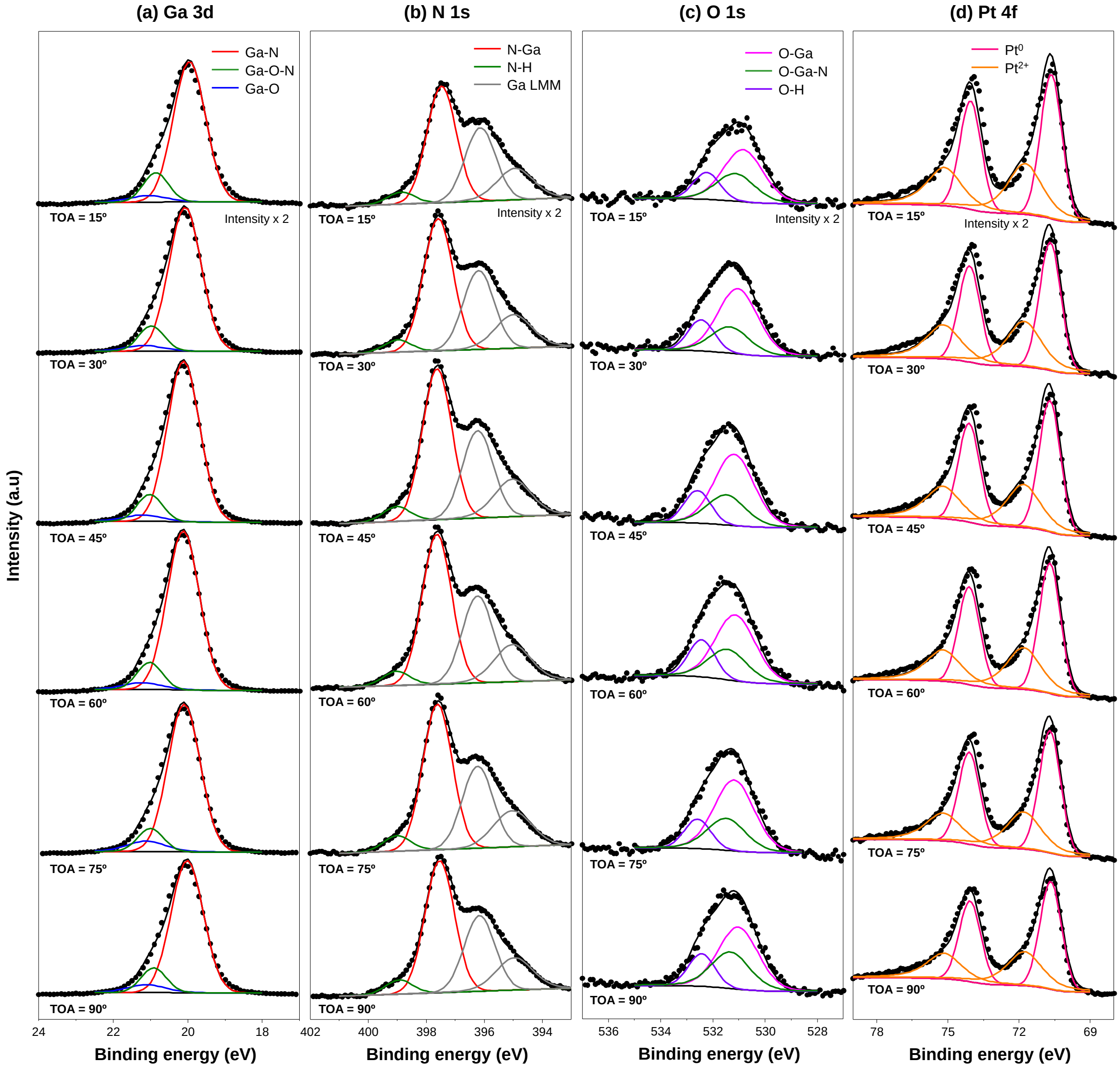

Figure S11. (a) Ga 3d, (b) N 1s, (c) O 1s, and (d) Pt 4f AR-XPS spectra of Pt/GaN/Si after 24 h reaction under 6.4 sun.

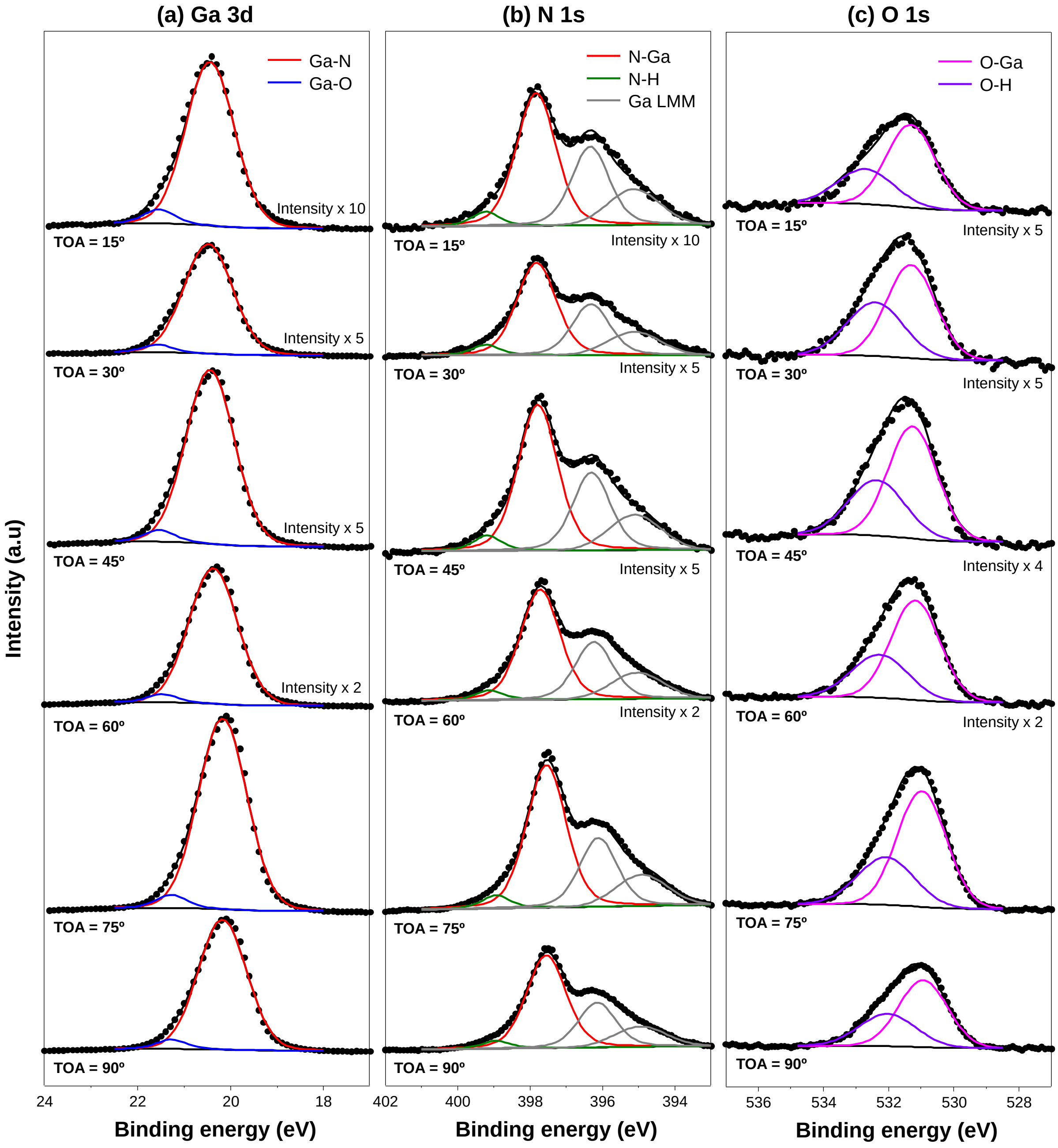

Figure S12. (a) Ga 3d, (b) N 1s, and (c) O 1s AR-XPS spectra of pristine GaN/Si.

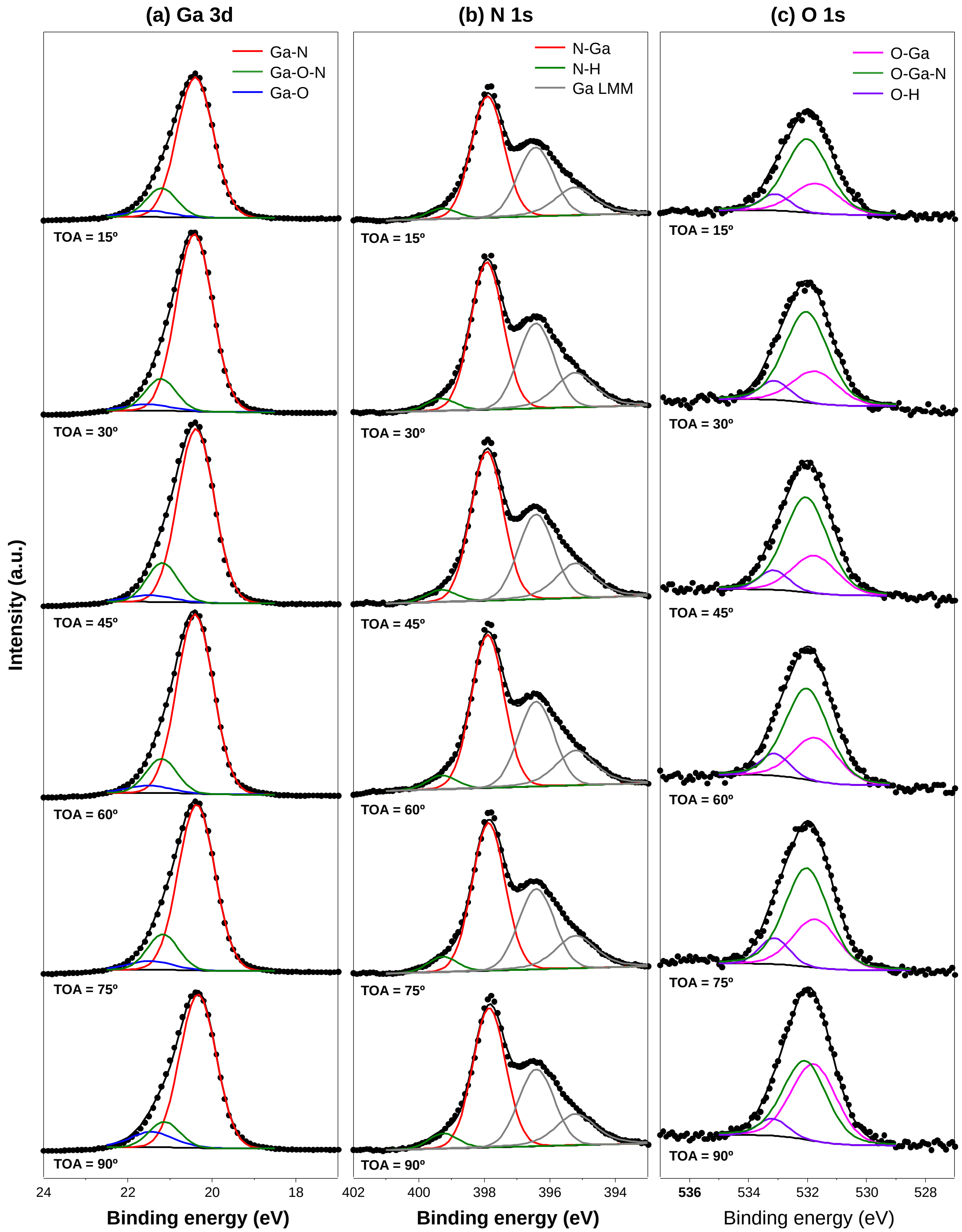

Figure S13. (a) Ga 3d, (b) N 1s, and (c) O 1s AR-XPS spectra of GaN/Si after 24 h reaction under 6.4 sun.

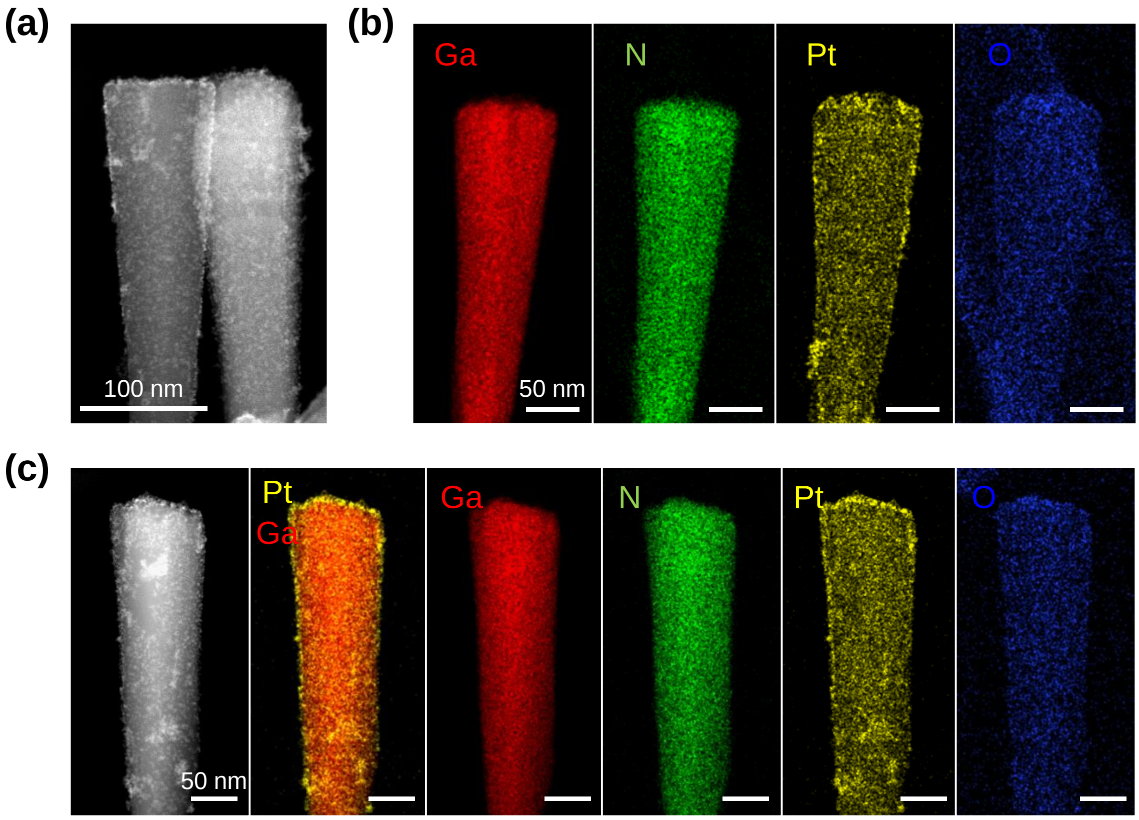

Figure S14. High-angle annular dark-field scanning transmission electron microscopy (HAADF-STEM) images and energy-dispersive X-ray spectroscopy (EDS) elemental maps of pristine Pt/GaN. (a-c) Three different regions of Pt/GaN NWs.

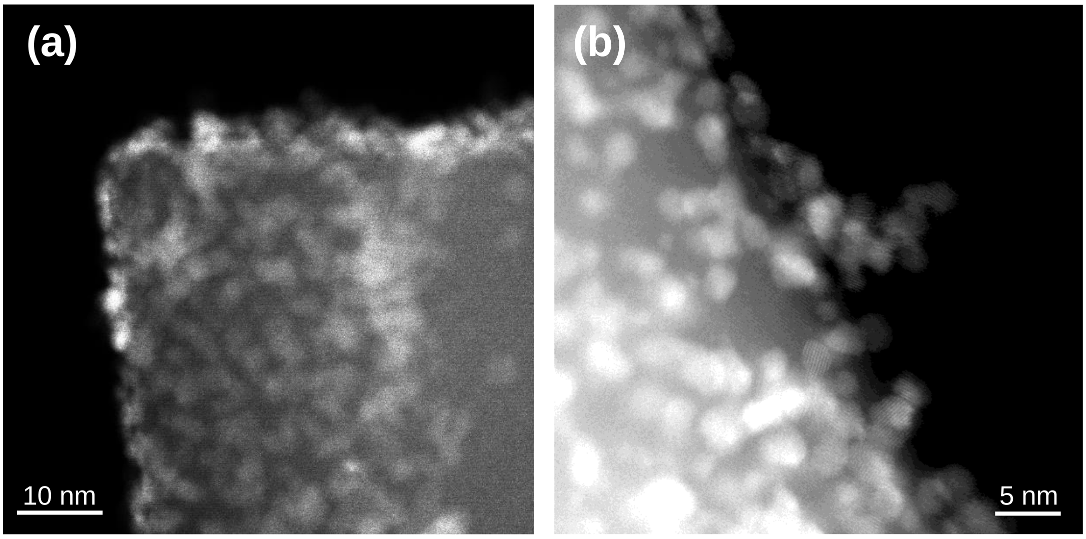

Figure S15. HAADF-STEM images of pristine Pt/GaN at different locations. Pt NPs showed bright Z-contrast.

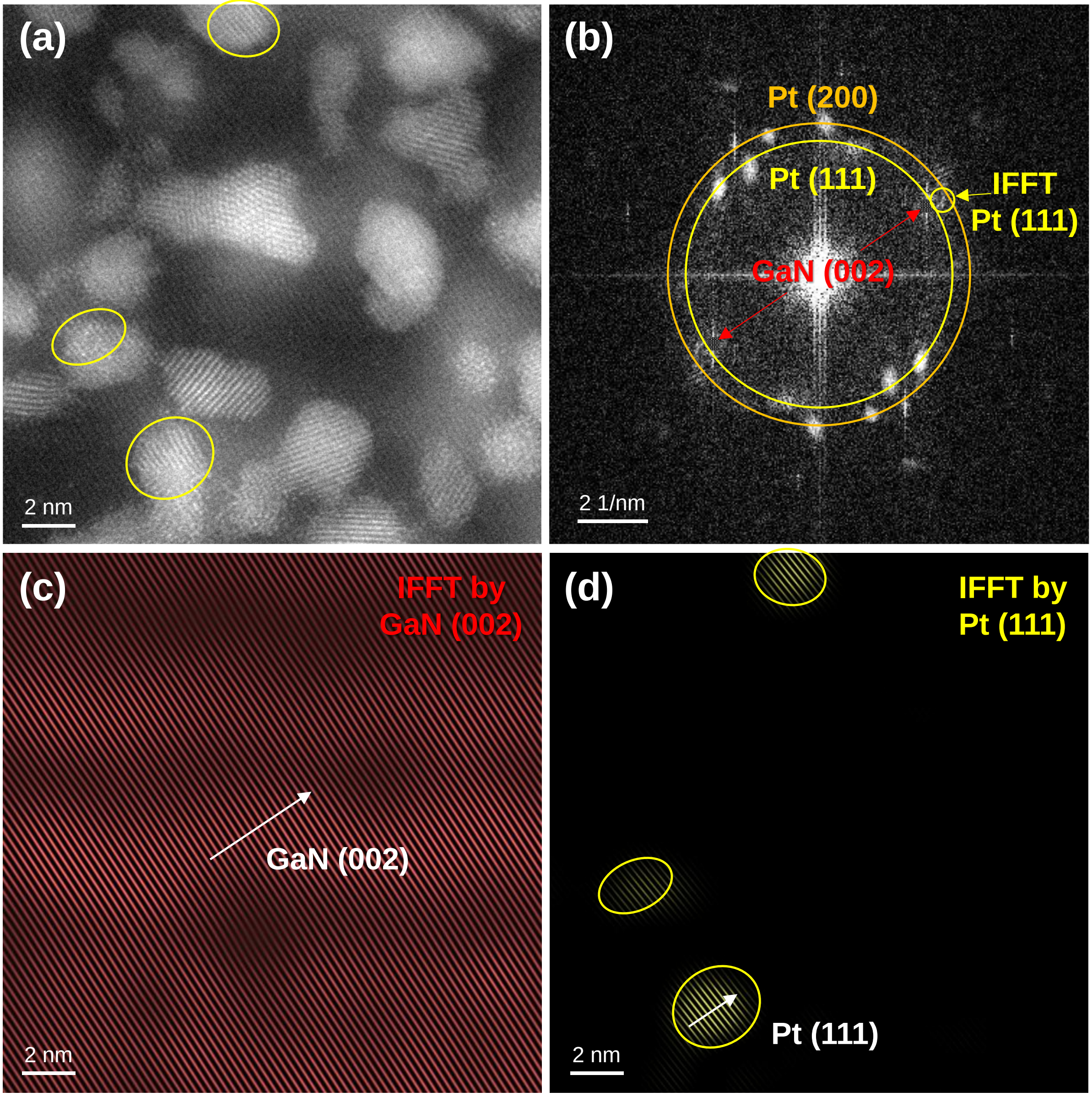

Figure S16. (a) HAADF-STEM image and (b) electron diffraction patterns of pristine Pt/GaN. Inverse Fourier-filtered images by masking (c) GaN (002) and (d) Pt (111) indicated in Figure S15b.

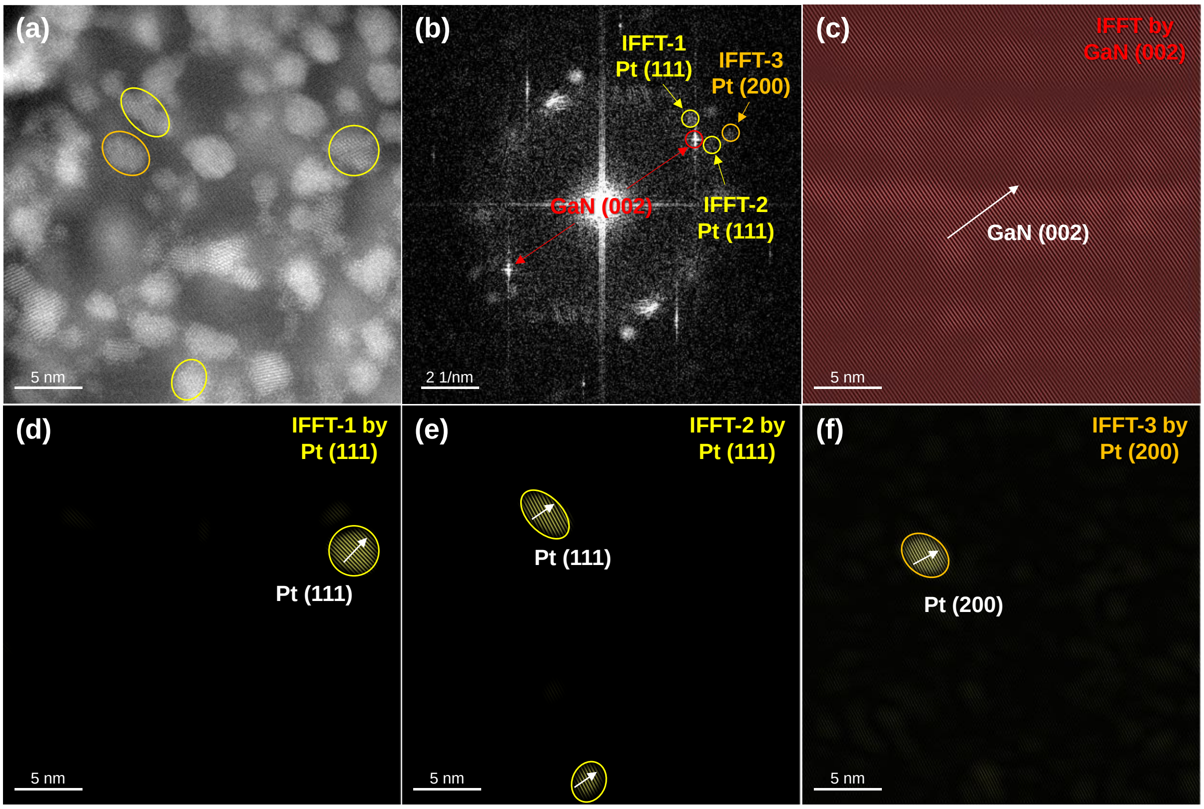

Figure S17. (a) HAADF-STEM image and (b) electron diffraction patterns of pristine Pt/GaN at different location. Inverse Fourier-filtered images by masking (c) GaN (002), (d,e) Pt (111), and (f) Pt (200) indicated in Figure S16b.

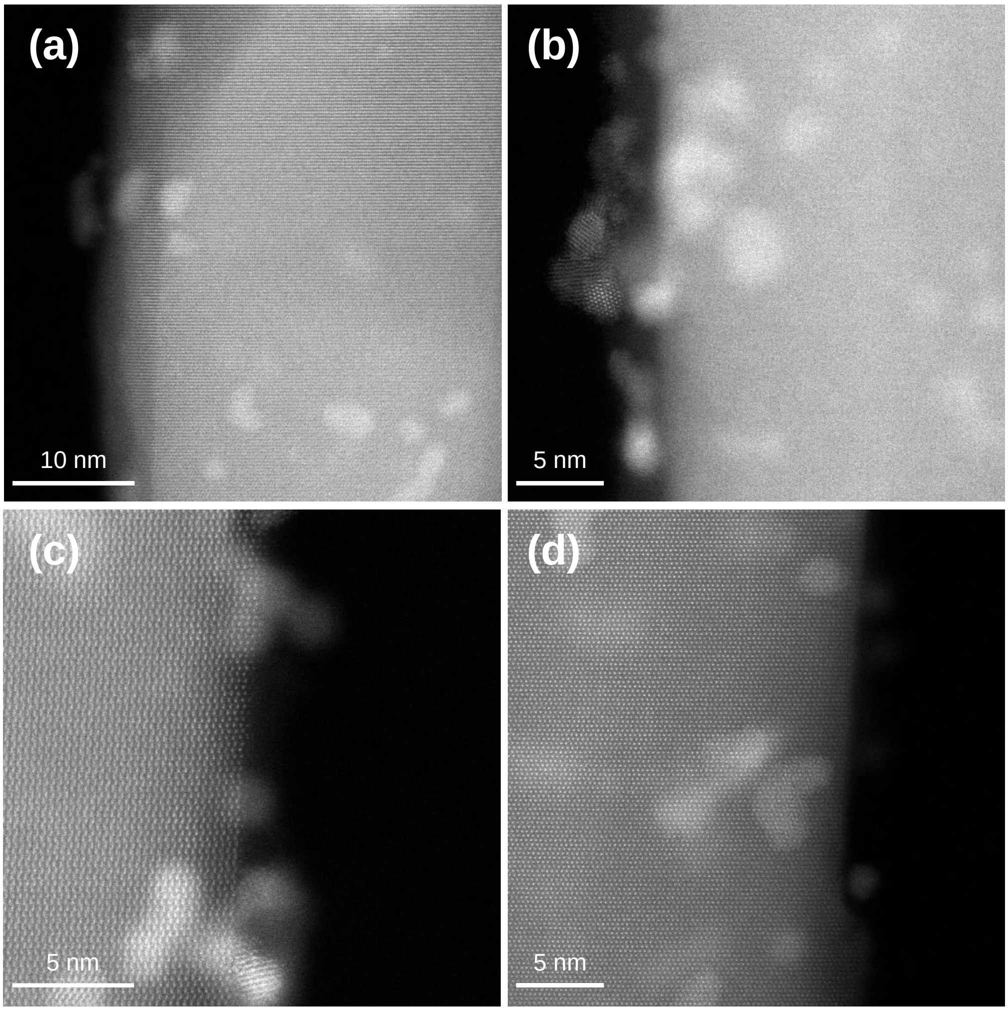

Figure S18. HAADF-STEM images of Pt/GaN after reaction for 24 h under concentrated solar light at 0 V_{RHE}. (a-d) Four different locations were characterized.

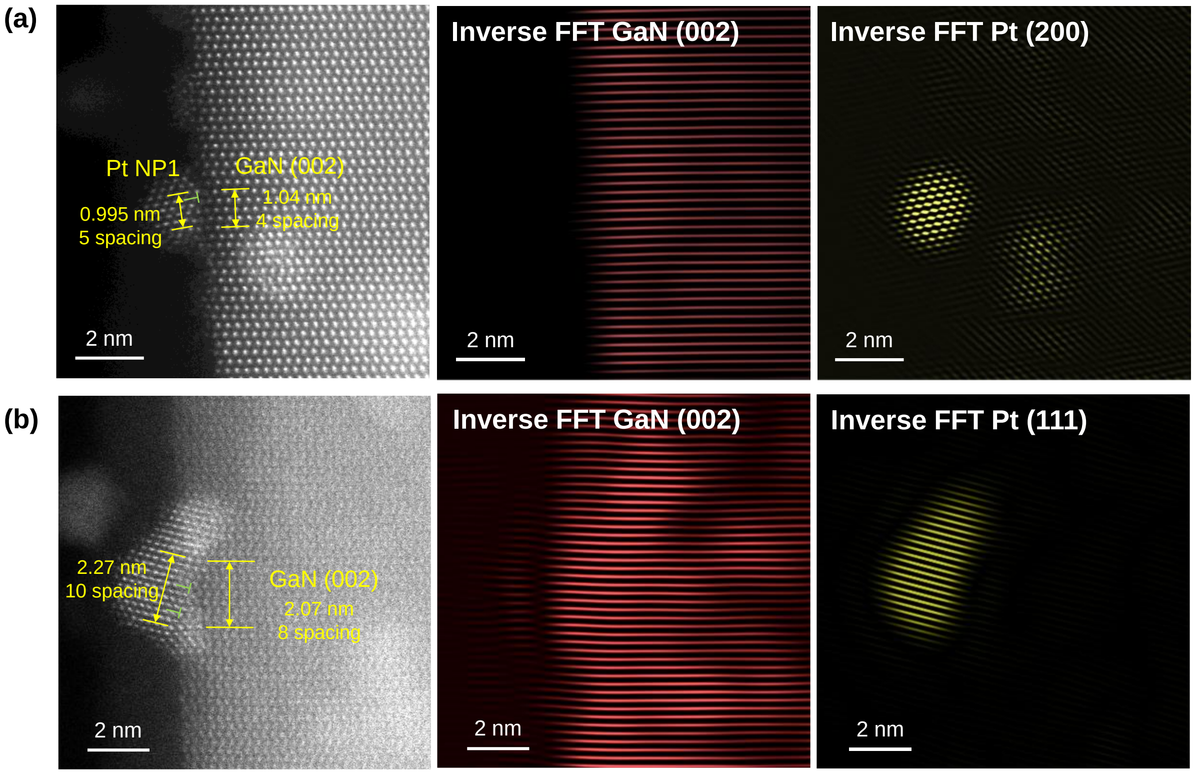

Figure S19. High-resolution STEM images and inverse Fourier-filtered images at two different locations. Specifically, the 5 lattice spacings of Pt (200) were aligned to the 4 lattice spacings of GaN (002) with an edge dislocation propagating in Pt NP1 (Figure S19a) and 10 lattice spacings of Pt (111) aligned to the 8 spacings of GaN (002) with two dislocations in Pt NP (Figure S19b).

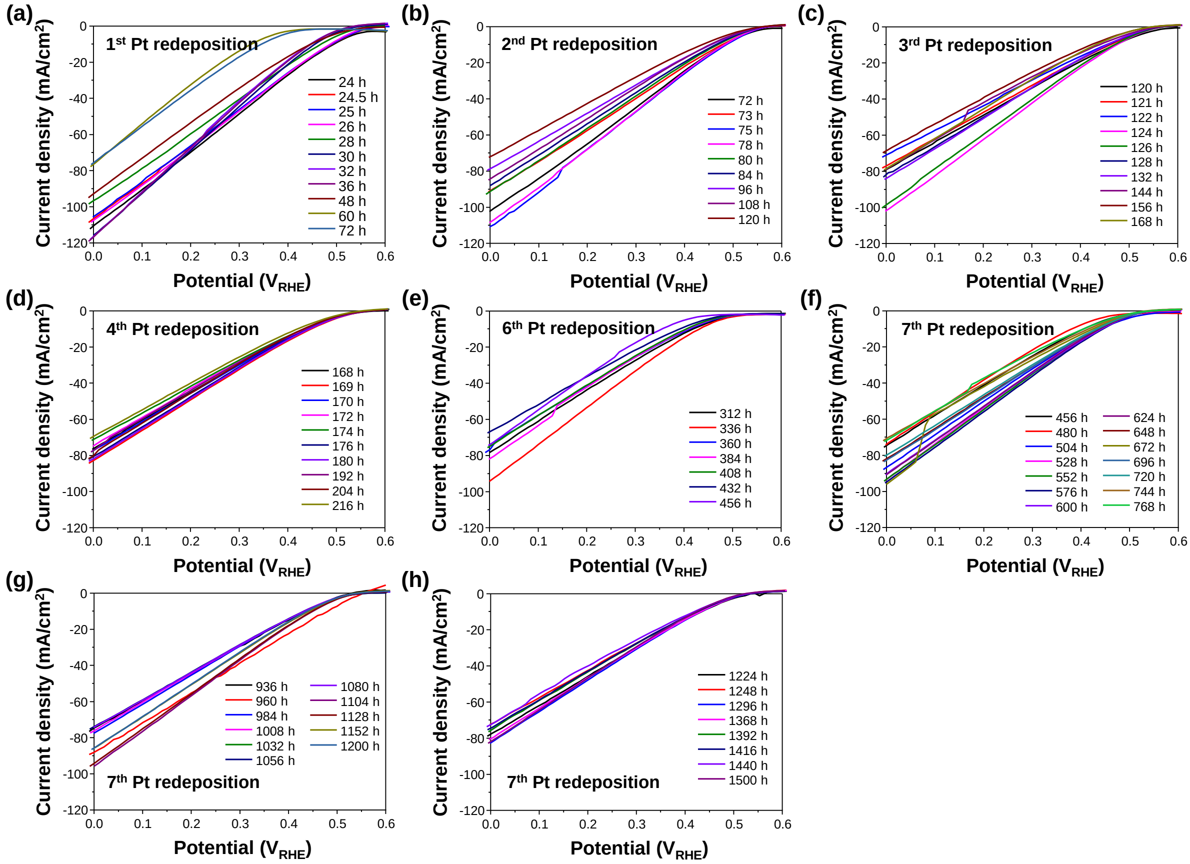

Figure S20. (a-h) LSV curves of Pt/GaN/Si photoelectrode after each period of reaction and Pt redeposition. Pt was redeposited for 7 times during 1500 h stability test.

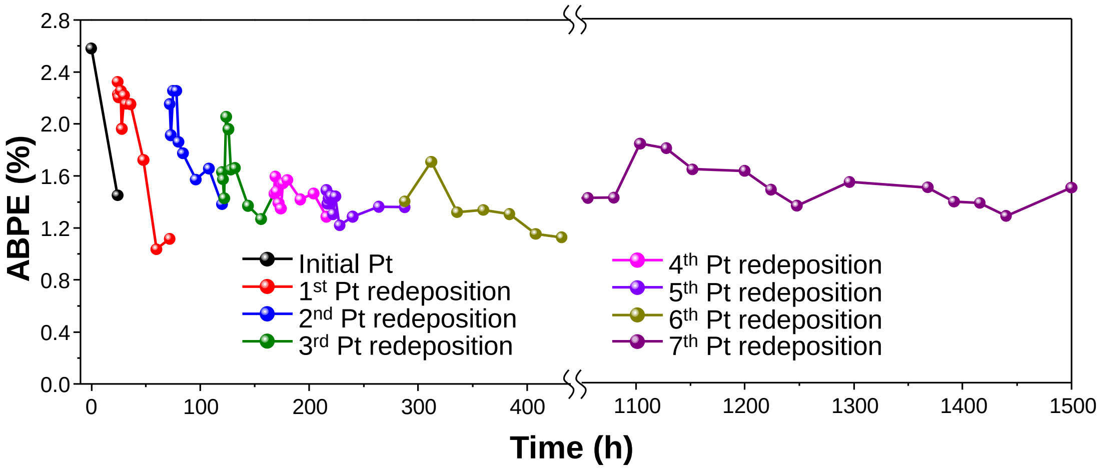

Figure S21. Plots of ABPE of Pt/GaN/Si over reaction time. The stability was tested over 1500 h under 6.4 sun light intensity. ABPE was eventually stabilized to ~1.5% during the concentrated solar light experiment.

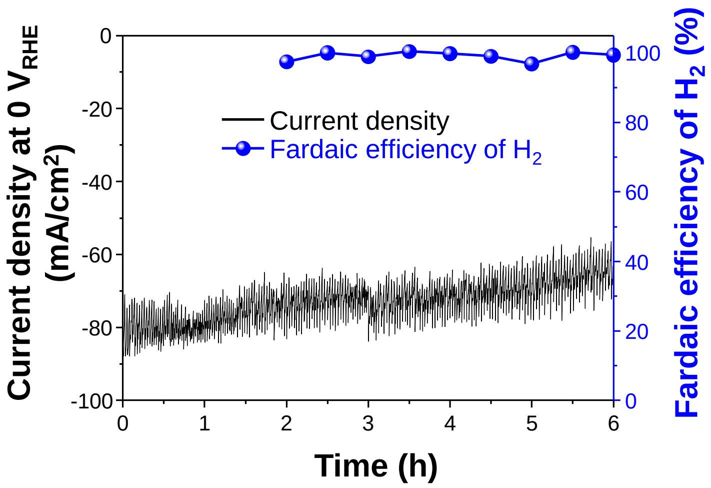

Figure S22. Current density and H_2 faradaic efficiency of Pt-redeposited GaN/Si photoelectrode at 0 V_{RHE} under concentrated solar light (6.4 sun). Near-unity H_2 faradaic efficiency was demonstrated.

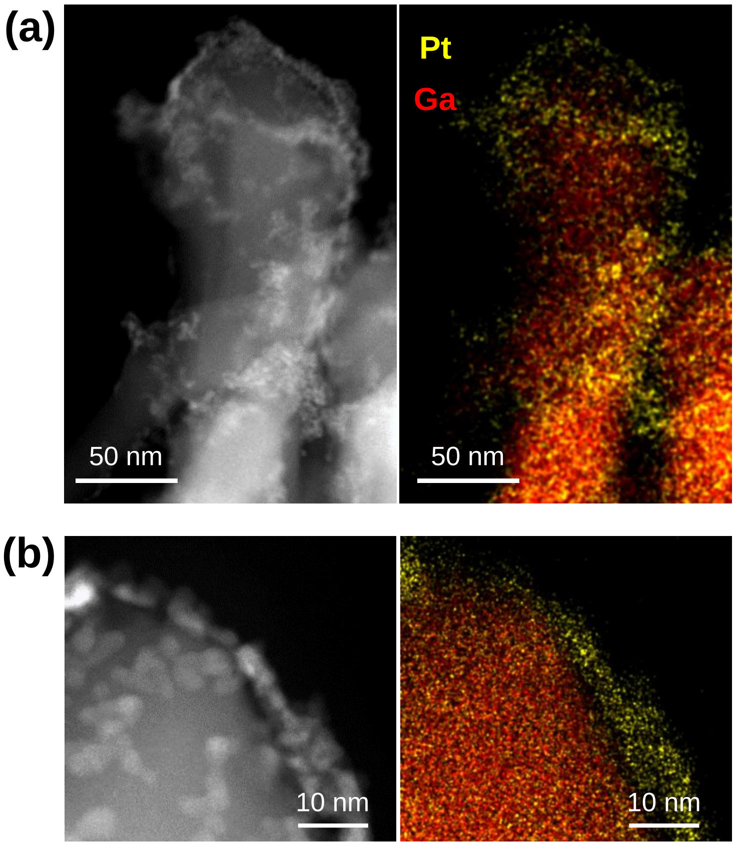

Figure S23. HAADF-STEM images and EDS elemental maps of Pt/GaN after 288 h reaction under concentrated solar light at 0 V_{RHE}.

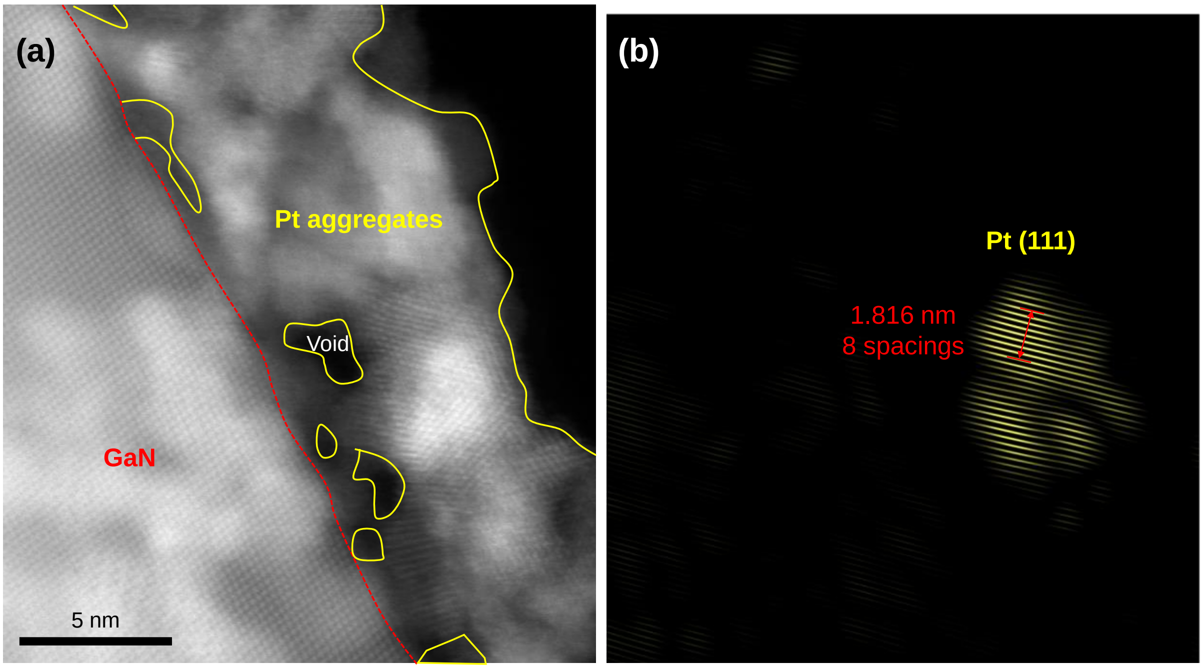

Figure S24. (a) High magnification HAADF-STEM image and (b) Inverse Fourier-filtered image by masking Pt (111) of Pt/GaN after reaction for 288 h under concentrated solar light at 0 V_{RHE}.

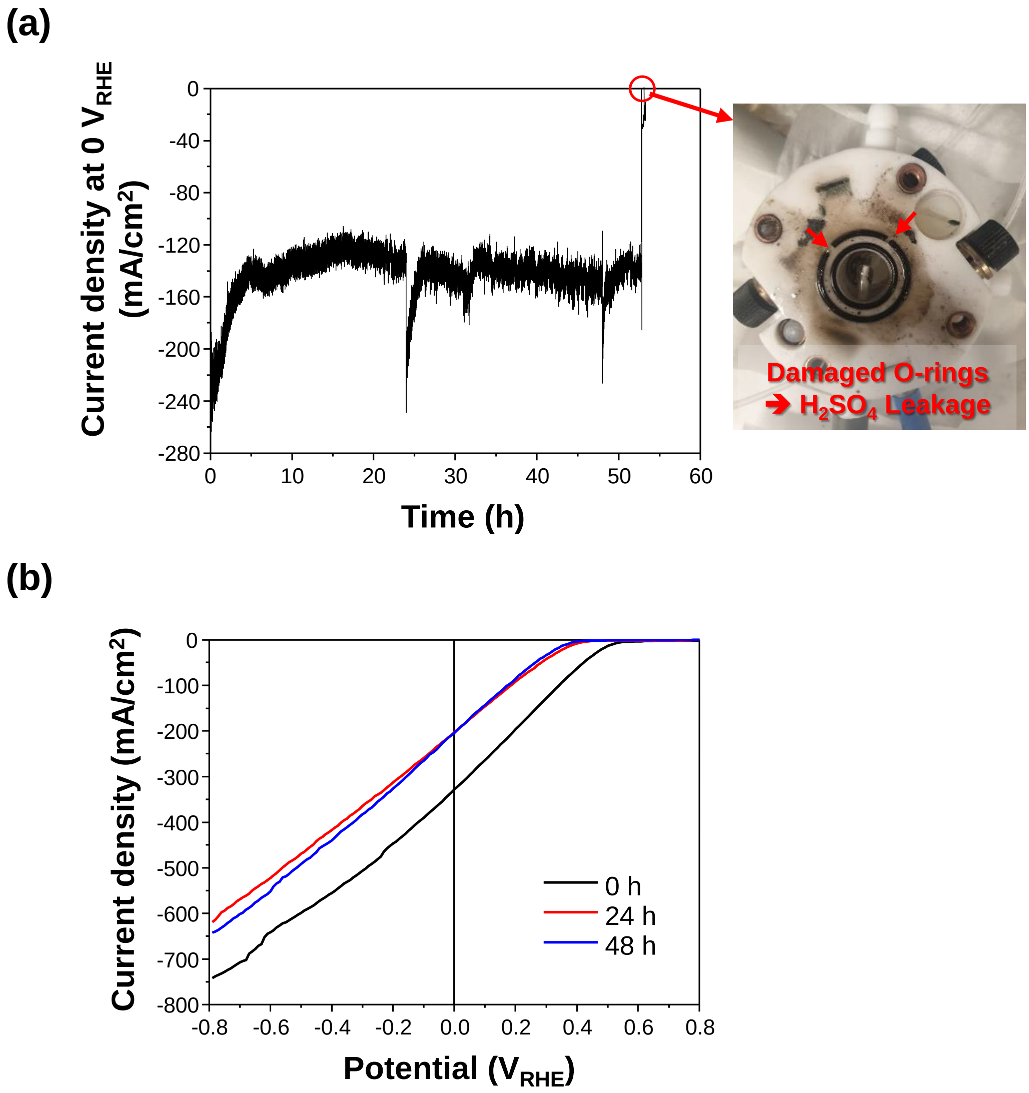

Figure S25. Highly concentrated solar light hydrogen evolution reaction under 40 sun. (a) J_{ph} at 0 V_{RHE} and (b) linear sweep voltammetry curves of Pt/GaN/Si photoelectrode. The current density at 0 V_{RHE} decreased from ~260 to ~140 mA/cm^2 during the initial reaction and then stabilized until 48 h. After ~52 h of reaction, intense ultraviolet light damaged the rubber O-rings of the reactor as shown in the inset photograph. This caused electrolyte leakage in the cathodic compartment and resulted in a rapid drop in J_{ph}.

Table S1. Performance and stability comparison of photoelectrodes for H_2 evolution reaction.

Photoelectrode	Light intensity	J at 0 V_{RHE}	Reaction time	J/J_0 after stability	Electrolyte	Ref.
Pt/GaN/n^+-p Si	6.4 sun	~120 mA/cm^2	1500 h	~75%	0.5 M H_2SO_4	**This work**
Pt/GaN/n^+-p Si	1 sun	~35 mA/cm^2	3000 h	100%	0.5 M H_2SO_4	[1]
SiO_x/Pt/SiO_2/p-Si	1 sun	10 mA/cm^2	12 h	100%	0.5 M H_2SO_4	[2]
Pt/TiO_2/pn^+-Si	1 sun	22 mA/cm^2	72 h	100%	1 M $HClO_4$	[3]
TiO_2/Pt/n^+-p Si	1 sun	~35 mA/cm^2	168 h	100%	1 M $HClO_4$	[4]
PtRu-GaInP/AlInP/GaInP-GaInP/GaInAs	1 sun	~13 mA/cm^2	1 h	85%	3 M H_2SO_4	[5]
Rh/AlInP-GaInP/GaInAs	1 sun	~15 mA/cm^2	40 h	~50%	1 M $HClO_4$	[6]
Pt/GaN/3J $GaInP_2$/GaAs/Ge	1 sun	~11 mA/cm^2	80 h	~80%	0.1 M H_2SO_4	[7]
PAAM/Pt/TiO_2/Sb_2Se_3	1 sun	19 mA/cm^2	100 h	70%	0.1 M H_2SO_4	[8]
Pt/TiO_2/Sb_2Se_3	1 sun	~13 mA/cm^2	2 h	60%	0.1 M H_2SO_4	[9]
Pt/TiO_2/CdS/Sb_2Se_3	1 sun	~9 mA/cm^2	10 h	85%	0.5 M Na_2SO_4 0.25 M Na_2HPO_4 0.25 M NaH_2PO_4	[10]
Pt/HfO_2/CdS/Cu_2ZnSnS_4	1 sun	~12 mA/cm^2	10 h	100%	0.2 M Na_2HPO_4 /NaH_2PO_4	[11]
Pt/TiO_2/AZO/Cu_2O	1 sun	~6 mA/cm^2	20 h	90%	0.5 M Na_2SO_4 0.1 M KH_2PO_4	[12]
NiMo/TiO_2/Ga_2O_3/Cu_2O	1 sun	~10 mA/cm^2	100 h	~80%	0.5 M Na_2SO_4 0.1 M KH_2PO_4	[13]
RuO_x/TiO_2/Cu_2O	1 sun	~9 mA/cm^2	55 h	70%	0.5 M Na_2SO_4 0.1 M KH_2PO_4	[14]

References

[1] S. Vanka, K. Sun, G. Zeng, T. A. Pham, F. M. Toma, T. Ogitsu, Z. Mi, *J. Mater. Chem. A* **2019**, *7*, 27612.

[2] N. Y. Labrador, X. Li, Y. Liu, H. Tan, R. Wang, J. T. Koberstein, T. P. Moffat, D. V. Esposito, *Nano Lett.* **2016**, *16*, 6452.

[3] B. Seger, T. Pedersen, A. B. Laursen, P. C. Vesborg, O. Hansen, I. Chorkendorff, *J. Am. Chem. Soc.* **2013**, *135*, 1057.

[4] R. Fan, W. Dong, L. Fang, F. Zheng, M. Shen, *J. Mater. Chem. A* **2017**, *5*, 18744.

[5] J. Gu, J. A. Aguiar, S. Ferrere, K. X. Steirer, Y. Yan, C. Xiao, J. L. Young, M. Al-Jassim, N. R. Neale, J. A. Turner, *Nat. Energy* **2017**, *2*, 1.

[6] M. M. May, H.-J. Lewerenz, D. Lackner, F. Dimroth, T. Hannappel, *Nat. Commun.* **2015**, *6*, 8286.

[7] Y. Wang, J. Schwartz, J. Gim, R. Hovden, Z. Mi, *ACS Energy Lett.* **2019**, *4*, 1541.

[8] J. Tan, B. Kang, K. Kim, D. Kang, H. Lee, S. Ma, G. Jang, H. Lee, J. Moon, *Nat. Energy* **2022**, *7*, 537.

[9] W. Yang, J. Ahn, Y. Oh, J. Tan, H. Lee, J. Park, H. C. Kwon, J. Kim, W. Jo, J. Kim, *Adv. Energy Mater.* **2018**, *8*, 1702888.

[10] L. Zhang, Y. Li, C. Li, Q. Chen, Z. Zhen, X. Jiang, M. Zhong, F. Zhang, H. Zhu, *ACS Nano* **2017**, *11*, 12753.

[11] D. Huang, K. Wang, L. Yu, T. H. Nguyen, S. Ikeda, F. Jiang, *ACS Energy Lett.* **2018**, *3*, 1875.

[12] J. Azevedo, L. Steier, P. Dias, M. Stefik, C. T. Sousa, J. P. Araújo, A. Mendes, M. Grätzel, S. D. Tilley, *Energy Environ. Sci.* **2014**, *7*, 4044.

[13] L. Pan, J. H. Kim, M. T. Mayer, M.-K. Son, A. Ummadisingu, J. S. Lee, A. Hagfeldt, J. Luo, M. Grätzel, *Nat. Catal.* **2018**, *1*, 412.

[14] J. Luo, L. Steier, M.-K. Son, M. Schreier, M. T. Mayer, M. Grätzel, *Nano Lett.* **2016**, *16*, 1848.
